# *Peltaster fructicola* genome reveals evolution from an invasive phytopathogen to an ectophytic parasite

**DOI:** 10.1038/srep22926

**Published:** 2016-03-11

**Authors:** Chao Xu, Huan Chen, Mark L. Gleason, Jin-Rong Xu, Huiquan Liu, Rong Zhang, Guangyu Sun

**Affiliations:** 1 State Key Laboratory of Crop Stress Biology in Arid Areas and College of Plant Protection, Northwest A&F University, Yangling, Shaanxi 712100, China; 2 Department of Plant Pathology and Microbiology, Iowa State University, Ames, Iowa 50011, USA; 3 Department of Botany and Plant Pathology, Purdue University, West Lafayette, Indiana 47907, USA

## Abstract

Sooty blotch and flyspeck (SBFS) fungi are unconventional plant pathogens that cause economic losses by blemishing the surface appearance of infected fruit. Here, we introduce the 18.14-Mb genome of *Peltaster fructicola*, one of the most prevalent SBFS species on apple. This undersized assembly contains only 8,334 predicted protein-coding genes and a very small repertoire of repetitive elements. Phylogenomics and comparative genomics revealed that *P. fructicola* had undergone a reductive evolution, during which the numbers of orphan genes and genes involved in plant cell wall degradation, secondary metabolism, and secreted peptidases and effectors were drastically reduced. In contrast, the genes controlling 1,8-dihydroxynaphthalene (DHN)-melanin biosynthesis and appressorium-mediated penetration were retained substantially. Additionally, microscopic examination of the surfaces of infected apple indicated for the first time that *P. fructicola* can not only dissolve epicuticular waxes but also partially penetrate the cuticle proper. Our findings indicate that genome contraction, characterized mainly by the massive loss of pathogenicity-related genes, has played an important role in the evolution of *P. fructicola* (and by implication other SBFS species) from a plant-penetrating ancestor to a non-invasive ectophyte, displaying a novel form of trophic interaction between plants and fungi.

Sooty blotch and flyspeck (SBFS) is a fungal disease complex that occurs worldwide on the fruit of apple, pear and persimmon as well as the leaves, stems and fruit of many other cultivated crops and wild plants. SBFS pathogens include more than 80 species, primarily within the order Capnodiales (Dothideomycetes, Ascomycota)^1^. These fungi colonize the epicuticular wax layer of hosts, forming darkly pigmented mycelial mats and fruiting bodies but causing no cell damage due to the absence of cell wall penetration. Such superficial dark-colored blemishes can lead to downgrading of fruit for fresh-market sale, which results in substantial economic losses^2^.

*Peltaster fructicola* Johnson, Sutton et Hodges, first described in North Carolina in 1996^3^, is a widely prevalent component of the SBFS complex on apple (*Malus* × *domestica*) in North America^2^, Europe^4^ and Asia^5^. It also causes SBFS signs on fruit of pawpaw (*Asimina triloba*) in Iowa, USA^6^ and crabapple (*Malus* × *micromalus*) and hawthorn (*Crataegus pinnatifida*) in China^7^. Although *P. fructicola* was classified within the Capnodiales along with most other SBFS species, the genus *Peltaster*, including another recently established species *P. cerophilus*, seems to be an isolated lineage^2^,^8^,^9^. Research on *P. fructicola* has focused mainly on its taxonomy, biogeography, pathogenicity, control by fungicides, and responses to temperature, humidity, and nutrients^10^,^11^,^12^,^13^,^14^,^15^. However, no investigations have probed this economically important plant pathogen, or other SBFS species, at the genome level.

SBFS pathogens are usually labeled as “epiphytic” because they occupy a surface-dwelling niche on many plant taxa^16^. Unlike most other functional groupings of plant pathogens such as biotrophs, hemibiotrophs, and necrotrophs, SBFS fungi neither invade living host cells nor actively absorb nutrients from them, but attach to plant surfaces and subsist primarily on tissue leachates^15^. These specialized fungi also differ from free-living saprophytes and symbionts in that they generally colonize living plants but confer no benefits to their hosts. The genetic and biochemical nature of the adaptations that enable survival in this unique niche has not been elucidated, although high-throughput sequencing and genome analysis have yielded deep insights into a wide range of traits in many other fungal pathogens and symbionts^17^,^18^,^19^.

Here, we describe the genome sequence of *P. fructicola* and present the transcriptome data and microscopic observations of *P. fructicola* during apple fruit surface colonization. Two main questions were addressed in this work: 1) what genomic features underpin the adaptation of SBFS pathogens to their epicuticular niche? 2) Under the hypothesis that SBFS fungi arose from invasive plant parasites^20^, what evolutionary mechanisms led to development of a so-called “epiphyte”?

## Results and Discussion

### Genome sequencing and general features

The genome of *P. fructicola* LNHT1506 was sequenced using an Illumina HiSeq 2000 sequencing platform. The total reads were 4,944 Mb in length, representing an approximately 253-fold sequence coverage. An 18.14-Mb draft genome was assembled and comprised 14 scaffolds (N50, 2.64 Mb). By calculating the sequencing depth with a frequency distribution of 21-base oligomers in usable reads (Supplementary Fig. S1)^21^, the genome size of *P. fructicola* was estimated to be 19.54 Mb, close to its actual assembled result. Only 308 ambiguous positions constituting three gaps were detected in the modified assembly with an average GC content of 51.9%. The Core Eukaryotic Genes (CEGs) Mapping Approach assessed the completeness of the *P. fructicola* genome to be 97.2% (241 out of 248 CEGs). A total of 8,334 protein-coding genes were predicted, over 97% of which were validated using mRNA sequences. Among these predicted proteins, 7,614 (91.4%) showed sequence similarities (BLASTP, cut-off e-value > 10^−6^) to the entries deposited in NCBI, 3,047 (36.6%) were mapped in the Kyoto Encyclopaedia of Genes and Genomes (KEGG) database, 3,856 (46.3%) were classified in the Clusters of Orthologous Groups (COG) database (Supplementary Fig. S2), and 5,202 (62.4%) were assigned to Gene Ontology (GO) terms (Supplementary Fig. S3).

Compared with 16 previously sequenced non-SBFS fungi (http://jgi.doe.gov/fungi) belonging to Capnodiales, the genome of *P. fructicola* is considerably smaller in both assembly size and gene number (Supplementary Fig. S4). However, the weak correlation (0.67) between the two variables suggests that gene count is not the primary factor in determining genome size, but rather that repeat content may play a larger role^22^. Therefore, we believe that *P. fructicola*’s extremely low repeat content (0.42%, far below that of most sequenced fungi) contributes more to its small assembly. Additionally, only nine rDNA tandem repeat units in *P. fructicola* were calculated (see Supplementary Notes), which is far less than that in animals (39 to 19,300), plants (150 to 26,048) and most well-studied fungi (dozens to hundreds), and meets the generally positive relationship between rDNA copy number and genome size^23^,^24^,^25^. Overall, the genome of *P. fructicola* presents an austerity characteristic, which may relate to its extreme living environment (prolonged exposure to dehydration, osmotic stress and ultraviolet radiation)^26^.

### Phylogeny and analysis of gene families

**Figure 1:**
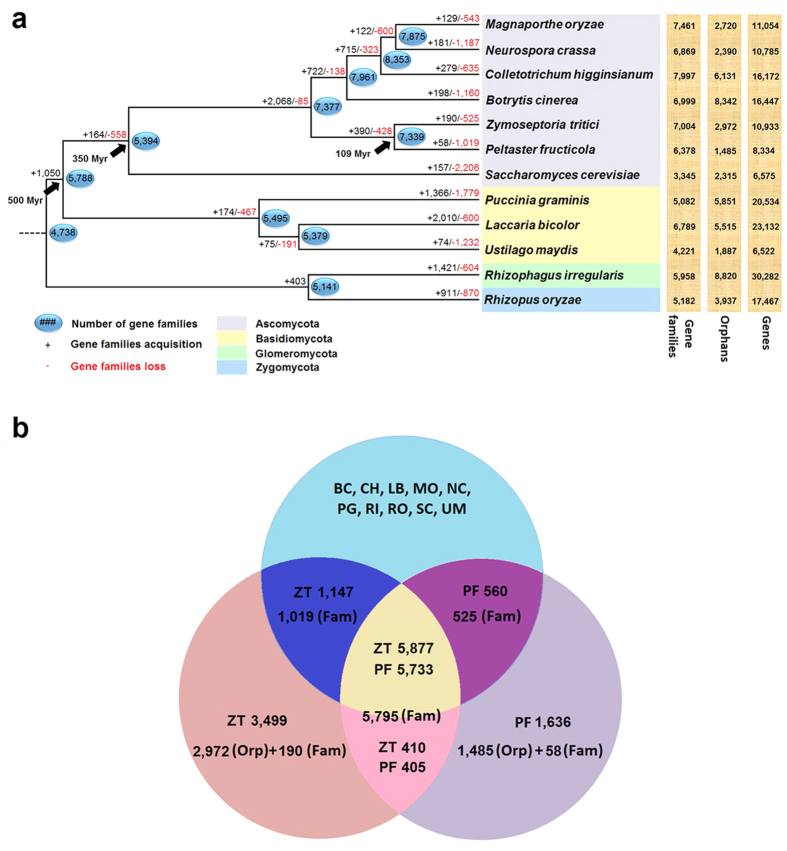
Phylogenetic relationship and analysis of gene families. (**a**) Predicted pattern of gain and loss of gene families in 12 representative fungal genomes used in this study. The numbers on the branches of the phylogenetic tree correspond to acquired (left, black), lost (right, red), and inferred ancestral (oval) gene families along each lineage by comparison with the putative pan-proteome. For each species, the number of gene families, orphan genes, and the total gene number are indicated on the right. The black arrows represent the divergence time of each two lineages with Myr used to abbreviate million years. (**b**) Venn diagram of the predicted genes and gene families in *P. fructicola* and *Z. tritici* versus those of 10 other fungal species. RF, ZT, BC, CH, LB, MO, NC, PG, RI, RO, SC, and UM respectively represent the abbreviations of the 12 fungal names shown in Fig. 1a. The numbers of genes (without mark), gene families (Fam) and orphan genes (Orp) are indicated in separate areas for *P. fructicola* and *Z. tritici*.

A genome-based maximum-likelihood (ML) tree was constructed using the amino acid sequences of 557 single-copy orthologous genes extracted from 12 fungi (Fig. 1a). All of its branches received 100% bootstrap values, implying a highly reliable topological structure. In this phylogram, *P. fructicola* clustered perfectly with *Zymoseptoria tritici* (formerly *Mycosphaerella graminicola*, and also in Capnodiales), and a large amount of gene families (5,795) shared by them suggested that these two relatives retain a substantial number of common characters (Fig. 1b). However, estimation of their divergence time reveals that they split from a common ancestor about 109 million years (Myr) ago, meaning that the two fungi are actually rather distantly related. This result strengthens findings of previous research suggesting that *P. fructicola* belongs to an early-diverging and independent lineage within the order Capnodiales^2^,^9^. Such a long-term separate evolution appears to have been accompanied by substantial differentiation. For example, *P. fructicola* experienced less acquisition of new gene families (58) and more loss of original families (1,019) than *Z. tritici* (190 new families and 525 lost families) (Fig. 1a), which suggests that fewer novel features were recruited whereas more original functions were abandoned by *P. fructicola* over the course of evolution. In addition, far fewer orphan genes (lacking homology to other sequences in the dataset^17^) were found in *P. fructicola* (1,485) than in *Z. tritici* (2,972) or the other selected fungi (1,887 to 8,820) (Fig. 1). This difference is noteworthy because some researchers proposed that such genes allow organisms to adapt to constantly changing ecological conditions^27^. Based on above analysis, we conclude that the genome of *P. fructicola* has undergone a reductive evolution, that is, loss of inherent genes and lack of novel genes.

The Dollo parsimony principle treated the 1,019 gene families from *Z. tritici* and the 525 gene families from *P. fructicola*, respectively, as the lost families of their opposites (Fig. 1). As a result, we could begin to discern which kinds of genes these two fungi have lost over the course of evolution by means of COG annotation. The result indicates that *P. fructicola* exceeds *Z. tritici* in the number of lost genes under nearly every specific function classification, especially those involved in amino acid transport and metabolism, carbohydrate transport and metabolism, and secondary metabolite biosynthesis, transport, and catabolism (difference of almost 50 genes) (Supplementary Fig. S5). Similarly, species-specific genes including orphans and new family genes in *P. fructicola* (1,636) and *Z. tritici* (3,499) were also annotated (Fig. 1b). Although only 10.3% and 11.7%, respectively, were assigned with functional items—possibly because these recently formed genes are short of homology—*P. fructicola* is comparatively deficient in three classifications: secondary metabolite biosynthesis, transport, and catabolism; amino acid transport and metabolism; and carbohydrate transport and metabolism (difference of over 30 genes) (Supplementary Fig. S6). Further research is needed to elucidate the functions of these three categories of genes in understanding the ecological adaptations of *P. fructicola*.

### Striking deficiency of secreted peptidases and candidate effectors

**Figure 2:**
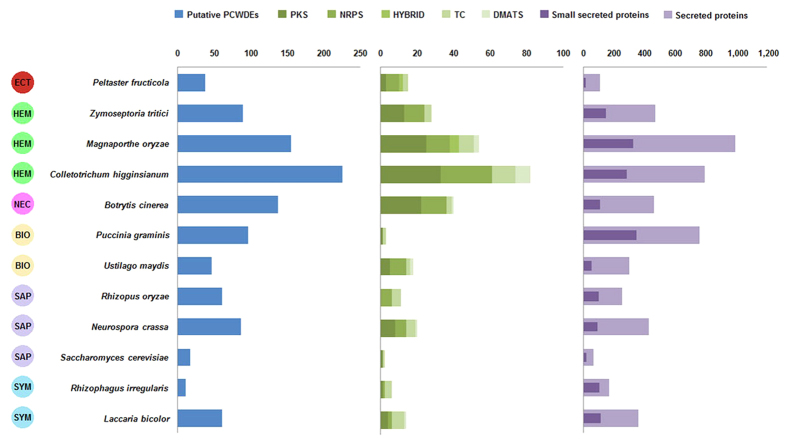
The numbers of genes encoding putative plant cell wall-degrading enzymes, key secondary metabolite synthetases and secreted proteins identified in the genomes of *P. fructicola* and 11 additional fungal species included in this study. The boxes on the left represent the life style of the selected organisms. ECT, ectophyte; HEM, hemibiotrophs; NEC, necrotroph; BIO, biotrophs; SAP, saprotrophs; SYM, symbionts. The colored bars representing the secondary metabolic enzymes are identified by the key at the top. PKS, polyketide synthases; NRPS, nonribosomal peptide synthases; TC, terpene cyclase; DMATS, dimethyl allyl tryptophan synthases; HYBRID, PKS-NRPS hybrids.

Secreted proteins are essential for fungi, especially for parasites and symbionts that participate in complex biochemical interaction with plants. These proteins include mainly lyases and hydrolases, which attack plant cell walls or degrade other complex carbon or nitrogen sources for nourishment (e.g., glucanases and proteases), and effectors, which facilitate infection (such as virulence factors or toxins) and/or triggering of defense responses (such as avirulence factors or elicitors) by manipulating host cell structures and functions^28^. *In silico* prediction identified 107 secreted proteins in *P. fructicola*, which exceeds only the number produced by *Saccharomyces cerevisiae* (62) but is far less than the other selected fungi (166 to 991) including phytopathogens, non-pathogenic saprophytes and symbionts (Fig. 2). Moreover, their proportion in the total gene number of *P. fructicola* (about 1.28%) is much lower than for most fungi that have been characterized previously (4% to 14%)^29^. The transcript profile revealed that only seven of the secreted protein-coding genes were both highly expressed and up-regulated (using a less stringent standard, ≥2-fold) on inoculated apple fruit surfaces; three were just highly expressed and nine were solely up-regulated (Supplementary Table S1). Diminution of the total secretome and its highly active genes accords with the fact of minimal biochemical interaction (e.g., breakdown of epicuticular waxes and digestion of fruit leachates) between *P. fructicola* and its hosts.

**Figure 3:**
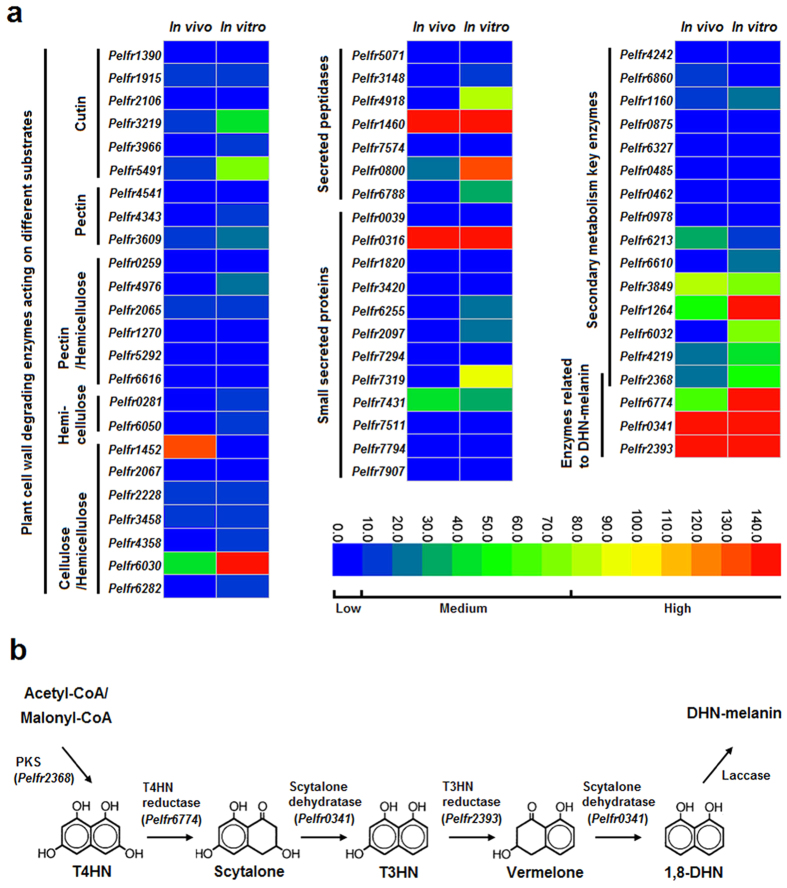
Functional analysis of the genes involved in adaptation of *P. fructicola* to its ecological niche. (**a**) Expression profiling of genes encoding secreted proteins and plant cell wall degrading enzymes and genes involved in biosynthesis of secondary metabolites. For the heatmaps, two columns represent different treatments, i.e., inoculation on apple fruit (*in vivo*) and growth on PDA media (*in vitro*), and each row is marked with the name of one gene (in italics). The colored scale bar of expression levels is divided into three grades: low (0 < RPKM < 10, including 0), medium (10 < RPKM < 80), and high (80 < RPKM). (**b**) Schematic representation of the fungal DHN-melanin biosynthesis pathway. Enzymes catalyzing the first five steps have been detected in the *P. fructicola* genome with their corresponding encoding genes listed in parentheses. RPKM, reads per kilobase per million mapped reads.

For both host-penetrating pathogens and saprophytes, secreted peptidases are essential in degrading plant tissues or other organic matter. For pathogens, these peptidases also block activation of host defenses. Proteolytic enzymes in the secretome of *P. fructicola*, however, were strongly reduced compared with non-SBFS species in the Capnodiales^22^, including only two aspartic endopeptidases (A01A subfamily), one zinc-metallopeptidase (M28E subfamily), two serine carboxypeptidases (S10 subfamily), one grifolisin-like peptidase (S53 subfamily) and one subtilisin-like peptidase (S8A subfamily) (Supplementary Table S2). Although the secreted zinc-metallopeptidase and subtilisin were once reported to serve as virulence factors by degrading hydroxyproline-rich glycoproteins (HRGPs), an important structural component within plant cell walls^30^,^31^,^32^, the fact that none of these seven proteinase-coding genes were up-regulated *in vivo* and five were expressed at very low levels (Fig. 3a) suggests that secreted peptidases make fairly limited contributions to the interaction between *P. fructicola* and host plants.

The numbers of small secreted proteins (SSPs), which are generally considered to be candidate effectors, ranged from 12 in *P. fructicola* to 343 in *Puccinia graminis* (Fig. 2). All the classical plant pathogens except *Ustilago maydis* possessed the largest numbers of SSPs, successively followed by symbionts and free-living saprophytes, while *P. fructicola* was in last place. As the effectors of classical plant pathogenic fungi are commonly cysteine-rich, functionally unknown and lacking in homology to proteins of other species^29^, we further filtered the SSPs of *P. fructicola*, finding that seven of them could be labeled as cysteine-rich, and eight had no functional annotations (PFAM domains); of the latter group, five lacked homology to proteins outside the genus *Peltaster* in GenBank (E-value < 0.001). Taken together, only one SSP produced by *P. fructicola* could meet all the three criteria for an effector (Supplementary Table S3). In addition, nearly all the SSP-coding genes of *P. fructicola* were expressed either minimally or not at all *in vivo* except two; one, although highly expressed, met none of the above criteria and the other, though expressed at intermediate level, encoded a protein with no cysteine residue (Fig. 3a; Supplementary Table S3). These results confirm that *P. fructicola* lacks effector-mediated plant-pathogen interaction, which is consistent with its inability to cause subcuticular infection.

### Loss of plant cell wall-degrading enzymes

Plant cell walls, which act as initial protective barriers against pathogen attack, are commonly composed of three classes of polysaccharides: cellulose, hemicellulose and pectin^33^. Accordingly, plant-pathogenic fungi evolved an array of plant cell wall degrading enzymes (PCWDEs) such as cellulase, hemicellulase and pectinase to depolymerize those components. Cutinase is also classed with the PCDWEs here because it plays a vital role in breaking down the plant cuticle, the outermost defense layer against pathogenic fungi^34^. A total of 38 putative PCWDEs were identified from the CAZymes of *P. fructicola*, which surpasses the numbers of only saprophytic *S. cerevisiae* and symbiotic *Rhizophagus irregularis*, but is far lower than classical plant pathogenic fungi excluding the biotrophic *U. maydis* (Supplementary Table S4; Fig. 2). The remaining saprophytic and symbiotic fungi were intermediate in the number of putative PCWDEs, indicating that they, despite non-aggressive lifestyles, still retain some cell wall-degrading abilities to decompose plant debris^35^. After manually eliminating the false positives, *P. fructicola* and its relative *Z. tritici* were left with 24 and 68 PCWDEs, respectively, which own credible functional annotations (Supplementary Table S5 and S6); the latter far surpasses the former in both kind and quantity of these enzymes. Moreover, only eight (33%) of the true PCWDEs in *P. fructicola* were identified as having secreting signals, whereas this proportion was 47% in *Z. tritici*. Compared with other plant pathogens and even some nonpathogenic species, *P. fructicola* has a severely reduced system for degrading plant cell walls, explaining why this fungus cannot penetrate host cells.

**Figure 4:**
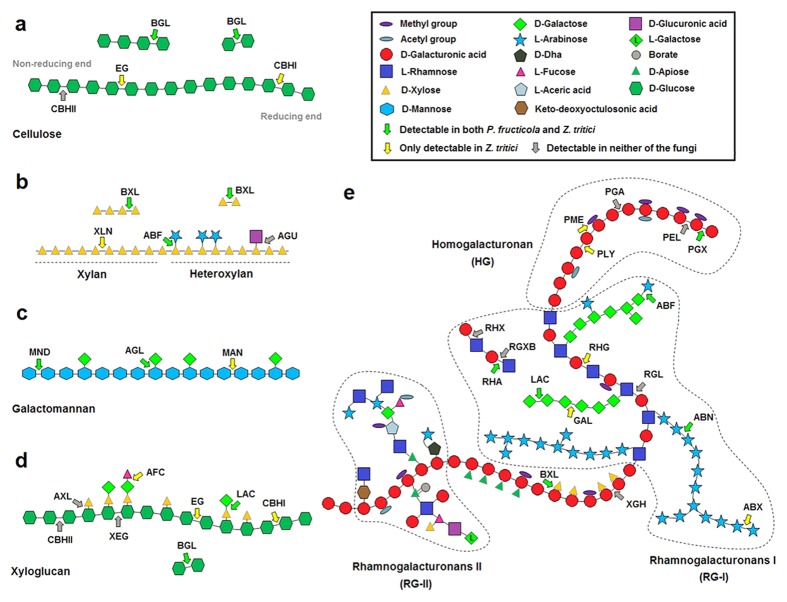
Schematic representation of plant cell wall polysaccharides and selected corresponding polysaccharide-degrading enzymes. (**a**) Cellulose; (**b**) Xylan and heteroxylan; (**c**) Galactomannan; (**d**) Xyloglucan; (**e**) Pectin. BGL, β-1,4-glucosidase; EG, β-1,4-endoglucanase; CBHI, cellobiohydrolase (reducing end); CBHII, cellobiohydrolase (nonreducing end); BXL, β-1,4-xylosidase; XLN, β-1,4-endoxylanase; ABF, α-arabinofuranosidase; AGU, α-glucuronidase; MND, β-1,4-mannosidase; AGL, α-1,4-galactosidase; MAN, β-1,4-endomannanase; AXL, α-xylosidase; XEG, xyloglucan β-1,4-endoglucanase; AFC, α-fucosidase; LAC, β-1,4-galactosidase; PGX, exopolygalacturonase; PEL, pectin lyase; PGA, endopolygalacturonase; PME, pectin methyl esterase; PLY, pectate lyase; ABF, α-arabinofuranosidase; RHG, rhamnogalacturonase; RHX, rhamnogalacturonan α-1,2-galacturonohydrolase; RGXB, rhamnogalacturonan α-L-rhamnopyranohydrolase; RHA, α-L-rhamnosidase; GAL, β-1,4-endogalactanase; RGL, rhamnogalacturonan lyase; ABN, endoarabinanase; ABX, exoarabinanase; XGH, endo-xylogalacturonan hydrolase; BXL, β-1,4-xylosidase.

We then compared *P. fructicola* and *Z. tritici* in terms of the specific roles of their true PCWDEs in degrading plant cell walls (Fig. 4). For fully hydrolyzing celluloses, three main cellulases are synergistic in activity: β-1,4-endoglucanase (EG) (EC 3.2.1.4), cellobiohydrolase (CBH; including CBHI and CBHII) (EC 3.2.1.176 and EC 3.2.1.91) and β-glucosidase (BGL) (EC 3.2.1.21). *Z. tritici* is equipped with all these cellulases, excluding CBHIs that release cellobioses from reducing ends of cellulose chains (Fig. 4a). By contrast, neither EGs (internal cleaving of cellulose chains) nor CBHs were detected in *P. fructicola*, and only the BGLs (for releasing of terminal glucoses from shorter oligosaccharides) could be found (Fig. 4a). For *P. fructicola*, this means a nearly complete absence of the ability to decompose celluloses.

More complex hydrolases are required for degrading hemicelluloses that consist of three major types of amorphous structures: xylan, galactomannan and xyloglucan. *P. fructicola* possesses β-1,4-xylosidases (BXLs) (EC 3.2.1.37) that release D-xyloses from non-reducing ends of xylooligosaccharides, but lacks β-1,4-endoxylanases (XLNs) (EC 3.2.1.8) that cleave xylan backbones into shorter oligomers. In contrast, *Z. tritici* possesses both the enzymes (Fig. 4b). Consequently, *P. fructicola* is unable to degrade xylans. Similarly, *P. fructicola* possesses β-1,4-mannosidases (MNDs) (EC 3.2.1.25) that release D-mannoses from terminal ends of galactomannans, but lacks β-1,4-endomannanases (MANs) (EC 3.2.1.78) that cleave galactomannans into mannooligosaccharides. However, *Z. tritici* possesses both of these enzymes (Fig. 4c). *P. fructicola* is therefore unable to degrade galactomannans. Moreover, xyloglucans cannot be depolymerized by *P. fructicola* because the backbone structure of xyloglucans is quite similar to that of celluloses (Fig. 4d).

Three classes of pectic polysaccharides have been characterized to date: homogalacturonan (HG), rhamnogalacturonan I (RG-I) and substituted galacturonan (including xylogalacturonan, apiogalacturonan and RG-II) (Fig. 4e)^36^. To degrade HGs, *Z. tritici* can first produce pectin methyl esterases (PMEs) (EC 3.1.1.11) to transform pectins to pectic acids by lowering the degree of methyl esterification, then exopolygalacturonases (PGXs) (EC 3.2.1.67) and pectate lyases (PLYs) (EC 4.2.2.2) are respectively applied to terminal hydrolysis and internal cleavage of pectic acids. In contrast, *P. fructicola*, which has only one PGX-coding gene, is apparently unable to decompose HGs. For decomposing RG-Is, *Z. tritici* can produce endorhamnogalacturonases (RHGs) (EC 3.2.1.171) to release oligosaccharides by the endohydrolysis of α-D-GalA-(1,2)-α-L-Rha glycosidic bonds, and use α-rhamnosidase (RHA) (EC 3.2.1.40) to hydrolyze terminal non-reducing α-L-rhamnose residues. However, *P. fructicola* has only one RHA-coding gene and therefore cannot digest RG-Is. Finally, Xylogalacturonan hydrolases (XGHs) (EC 3.2.1.–) that can specifically digest xylogalacturonans were never detected in either species, and little is known about the enzymes degrading apiogalacturonans and RG-IIs.

Both *P. fructicola* and *Z. tritici* had six predicted cutinase-coding genes, which indicates that they may own a similar ability to disrupt the carboxylic ester bonds within cutins, the main component of plant cuticle, resulting in the release of cutin monomers.

The above comparative analysis reveals that *P. fructicola* has lost nearly all the key enzymes involved in breaking down the backbone chains of major components of plant cell walls. Also, as for the available PCWDE-coding genes (excluding cutinases), only one was highly expressed and five were intermediately expressed *in vivo* (Fig. 3a). Therefore, *P. fructicola* is unable to break through plant cell walls and invade host tissues. *Z. tritici*, by contrast, exhibits a basic competency to degrade plant cell walls that *P. fructicola* lacks^37^. However, presence of the same number of cutinase-coding genes in these two fungi suggests that *P. fructicola* may retain the ability to degrade cuticles, especially considering that two of them were expressed at a moderate level *in vivo* and have secretion signals (Fig. 3a; Supplementary Table S5).

### Reduced secondary metabolism and effective melanin biosynthesis

Secondary metabolites (SMs, including mycotoxins, antibiotics and pharmaceuticals) are important compounds for colonization of specific ecological niches by many fungi. For example, SMs help saprophytes to compete for limited nutrients, minerals and water^38^, and more crucial to many parasites, they are used as weapons to weaken or even kill the hosts^39^. In *P. fructicola*, we found only 15 SM biosynthesis enzyme-coding genes that belong to four classes: polyketide synthase (PKS), non-ribosomal peptide synthetase (NRPS), polyketide synthase/non-ribosomal peptide synthetase hybrid (PKS-NRPS), and terpene cyclase (TC) (Supplementary Table S7). This number is far smaller than those of non-biotrophic plant pathogenic fungi and even slightly smaller than that of the biotrophic pathogen *U. maydis* (Fig. 2), in which SM genes should be diminished, probably because their existence compromises host survival^40^. Moreover, over half of the 15 SM genes presented poor transcriptional activity whether *in vivo* or *in vitro* (Fig. 3a). These findings conform to our expectation that pathogenicity-related SM genes are unnecessary for *P. fructicola*, which never causes observed physiological damage to host plant cells. We therefore suspect that the major role of the retained SMs in *P. fructicola* is more likely to resist other colonists that can occupy the same ecological niches. Venkatasubbaiah *et al.*^14^ identified four SMs produced by *P. fructicola* including trichothecolone, trichothecolone acetate, 6-methylsalicylic acid and 2,5-dihydroxybenzoic acid. Among them, trichothecolone acetate and 6-methylsalicylic acid were proved to have strong antifungal properties against *Botryosphaeria* spp. and *Colletotrichum* spp. *in vitro* – thus enabling *P. fructicola* to compete with microbial antagonists on the fruit surface.

One *PKS* gene (intermediately expressed) was annotated as a polyketide synthase that catalyzes the reaction from acetyl-CoA or malonyl-CoA precursors to 1,3,6,8-tetrahydroxynaphthalene (T4HN), which is the first step in the 1,8-dihydroxynaphtalene (DHN)-melanin biosynthesis process usually found in ascomycetes^41^. This dark pigment can enable fungi to resist extreme temperatures, desiccation, ionizing radiation, and heavy metal toxicity^42^. Such benefits probably make melanin one of the most important determinants for adaptation of *P. fructicola* and other SBFS fungi, which can tolerate long-term exposure to ambient environments. The genome of *P. fructicola* was therefore searched for the other enzymes involved in synthesis of DHN-melanin, using *Cochliobolus heterostrophus* as the model^43^. Orthologs of the T4HN reductase (*BRN2*), scytalone dehydratase (*SCD1*), and 1,3,8-trihydroxynaphthalene (T3HN) reductase (*BRN1*) were found in *P. fructicola* (Fig. 3b; Supplementary Table S8), and were moderately to highly expressed both *in vivo* and *in vitro* (Fig. 3a), verifying that a large amount of melanin is indeed required by this species to adapt to its environment.

### The colonization strategy of *P. fructicola* on apple fruit

**Figure 5:**
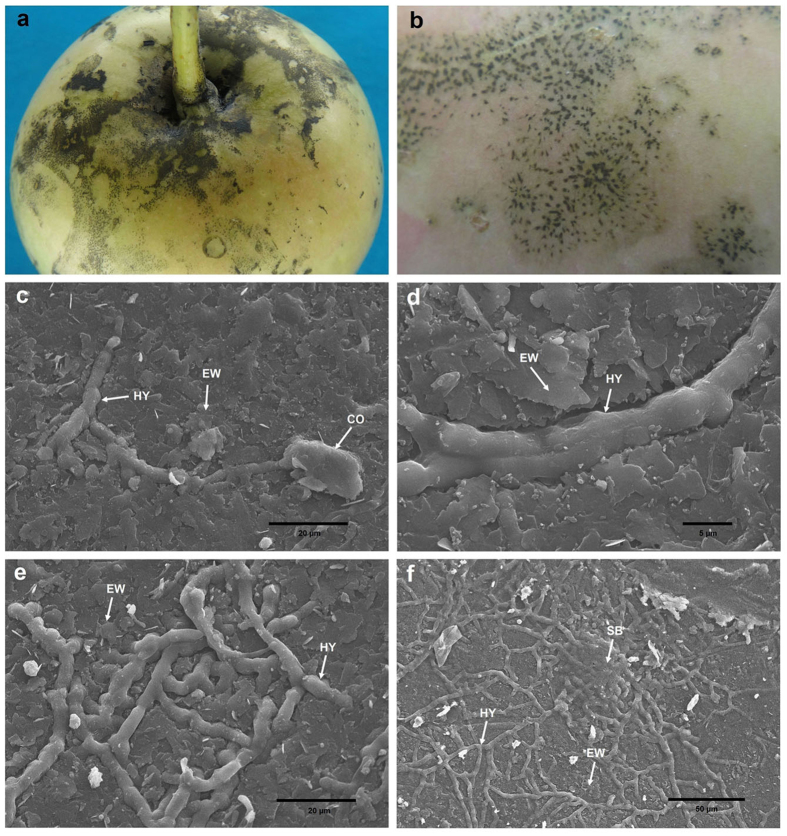
Photographs and scanning electron micrographs of *P. fructicola* on apple fruit. (**a,b**) SBFS signs caused by *P. fructicola* on apple fruit. (**c**) Germinating conidium and primary hypha partly submerged into the surface of epicuticular waxes. (**d,e**) Hyphae partly submerged into the surface of epicuticular waxes. (**f**) Sclerotium-like body and hyphae, both of which are partly submerged into the surface of epicuticular waxes. CO, conidium; HY, hypha; EW, epicuticular wax; SB, sclerotium-like body.

**Figure 6:**
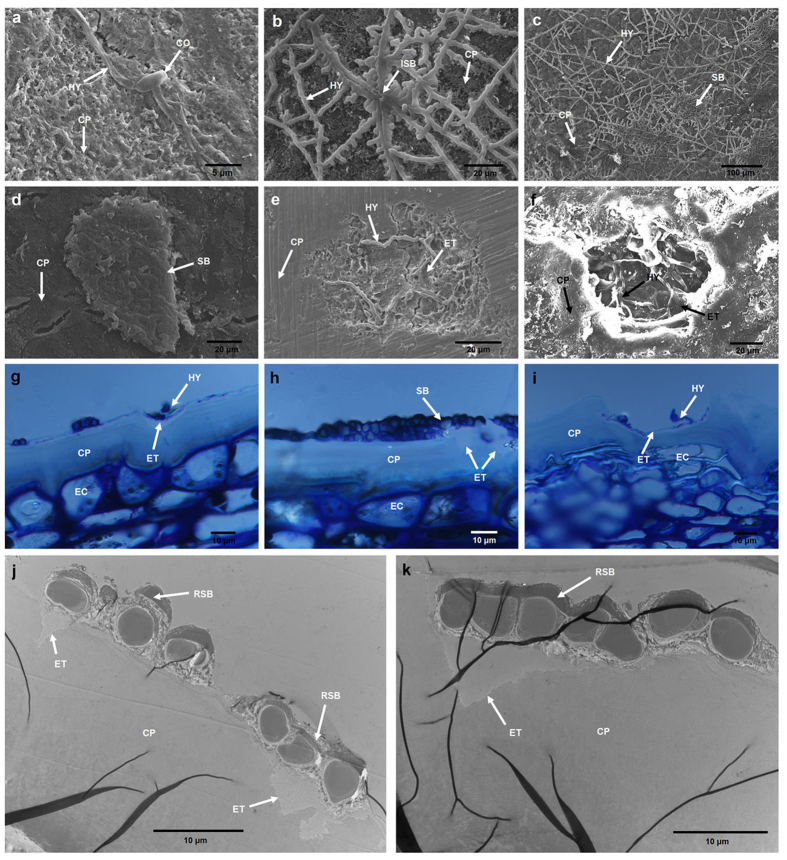
Scanning electron (**a–f**), optical (**g–i**) and transmission electron (**j,k**) micrographs of *P. fructicola* on apple fruit with epicuticular waxes removed. (**a**) Germinating conidium and and primary hypha. (**b**) Immature sclerotium-like body. (**c**) Sclerotium-like bodies and hyphae. (**d**) Sclerotium-like body without surrounding hyphae. (**e,f**) Degradation of the cuticle proper beneath sclerotium-like bodies. (**g–k**) Cross sections showing degradation of the cuticle proper beneath sclerotium-like bodies. CO, conidium; HY, hypha; SB, sclerotium-like body; ISB, immature sclerotium-like body; CP, cuticle proper; ET, eroded trace; RSB, remnant of sclerotium-like body; EC, epidermal cell.

**Figure 7:**
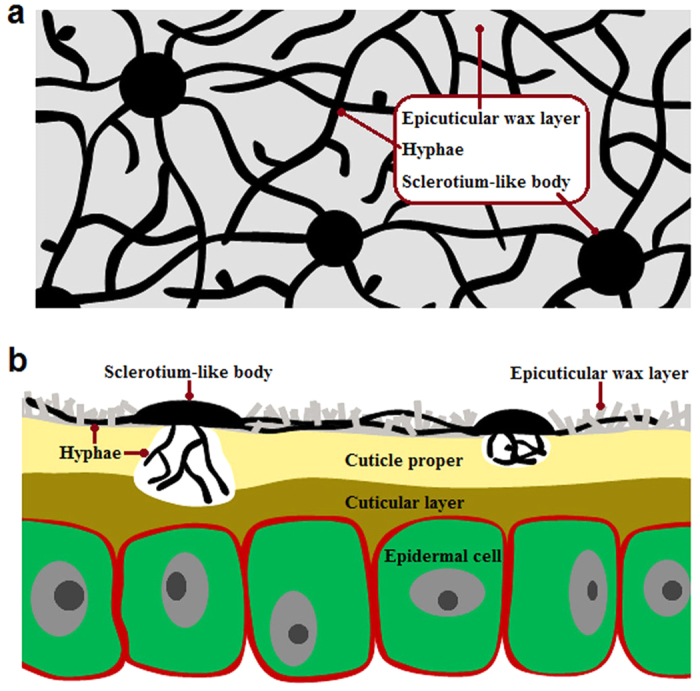
Inferred colonization pattern of *P. fructicola* on apple fruit. (**a**) Overhead view of *P. fructicola* growing on the apple fruit surface. (**b**) Sectional view of *P. fructicola* growing on the apple fruit surface.

In nature, SBFS pathogens are always restricted to the surfaces of hosts, either lying above the epicuticular wax layer or submerged into the wax crystals^1^,^16^. However, they have never been reported to penetrate the cuticle proper that underlies the epicuticular wax layer^44^,^45^,^46^, which results in uncertainty about where and how these organisms obtain sufficient nutrients for growth and reproduction. Belding *et al.*^16^ demonstrated that *P. fructicola* cannot metabolize or degrade epicuticular waxes whether *in vivo* or *in vitro*; it feeds on fruit leachates. Subsequently, *P. fructicola* was shown to germinate and grow on the sugars and amino acids exuded through the fruit cuticle, particularly during ripening under high relative humidity and moderate to warm temperatures^12^. Based on our microscopic observations of *P. fructicola* on apple fruit (Figs 5 and 6), we obtained somewhat different results from previous research and thereby inferred an ingenious colonization strategy adopted by this pathogen (Fig. 7; Supplementary Fig. S7).

Under scanning electronic microscopy (SEM), hyphae and sclerotium-like bodies (flattened, circular or irregular structures formed by tightly intertwining hyphae) were found to be partly submerged into the surface of the epicuticular wax layer of apple fruit, which provides evidence of degradation or absorption of waxes by *P. fructicola* (Fig. 5c–f). This result is contrary to the SEM study by Belding *et al.*^16^ which found no evidence of wax degradation. When cuticle samples without crystalline wax layers were observed, they showed no signs of further surface degradation around the hyphae and sclerotium-like bodies (Fig. 6a–d), which is not surprising if epicuticular wax deposits are always the limit of penetration by SBFS fungi. However, accidental removal of sclerotium-like bodies revealed different degrees of dissolution of the cuticle proper beneath these compact structures (Fig. 6e,f). This finding was confirmed by examining cross sections of the samples through both optical microscopy (OM) (Fig. 6g–i) and transmission electron microscopy (TEM) (Fig. 6j,k). In addition, our previous identification of the secretory cutinases in *P. fructicola* also implies that the species can potentially degrade the cuticle proper.

Nevertheless, we agree with Belding *et al.*^16^ that the main energy sources for *P. fructicola* are probably the sugar-rich fruit leachates rather than the components of epicuticular waxes such as ursolic acid and n-alkanes. For *P. fructicola*, the primary purposes of degrading the epicuticular waxes are therefore more likely to be stronger attachment to the fruit surface and enhanced access to fruit leachates. Degradation of the cuticle proper is presumed to aid in achieving the same goals. However, it is intriguing that such degradation seems to occur only below the sclerotium-like bodies rather than hyphae. Our guess is that the sclerotium-like bodies probably play a role similar to that of appressoria in classical plant pathogens, which provide not only degrading enzymes but also mechanical forces to aid penetration. Indirect evidence to support this speculation is that *P. fructicola* contains orthologs of most (even slightly more than *Z. tritici*) of the genes involving autophagy^47^, ROS and four key signaling pathways^48^: the cyclic AMP-protein kinase A (cAMP-PKA) pathway, the MAP kinase (MAPK) pathway, the cell wall integrity MAPK pathway and the osmoregulation pathway, which have been reported to regulate and control the appressorium-mediated penetration in *Magnaporthe oryzae* (Supplementary Table S9).

The above colonization strategy can help *P. fructicola* to absorb as much nutrition as possible from its hosts in the absence of key PCWDEs used for penetrating epidermal cell walls. We speculate that further research may reveal similar colonization patterns across the range of SBFS fungi. Therefore, the term “epiphytic”, which generally indicates that only physical support is provided by host plants^49^, seems unsuitable to accurately describe these organisms; instead, “ectophytic”, which places more emphasis on the external parasitism (i.e., extraction of nutrients exuded from hosts)^50^, should be a better choice.

### Evolutionary pattern of SBFS fungi

A review by Spanu^51^ on the genomics of obligate and nonobligate biotrophs posited that all phytopathogenic fungi and oomycetes have a biotrophic infection phase, and therefore the continuous trophic spectrum takes shape from necrotrophs at one end, through hemibiotrophs and nonobligate biotrophs, to obligate biotrophs at the other end without hard and fast boundaries. According to this author, formation of such trophic (or pathogenic) types should be attributed to preadaptation of saprotrophic decomposers of plant-derived biomass rather than phyletic evolution. He concluded that saprophytes, under suitable conditions, evolved into the ancestral plant pathogens with a certain biotrophic ability; further adaptations might then lead to necrotrophy or obligate biotrophy (two extremes of the trophic spectrum), which were respectively accompanied by the expansion and diversification of genes involved in secondary metabolic phytotoxins and plant cell wall degradation, and the loss of primary metabolic pathways and carbohydrate depolymerases.

However, evolutionary origin of the SBFS fungi, with a distinctly different lifestyle, was never mentioned in his work. Ismail *et al.*^20^, based on ancestral state reconstruction using the phylogenetic trees of two genes (*LSU* and *RPB2*), provided evidence that the major SBFS lineages actually evolved from plant-parasitic ancestors. This interpretation is also supported by our phylogenomic analysis showing that *P. fructicola* shared a common ancestor with the hemibiotrophic plant pathogen *Z. tritici*, dating back to about 100 Myrs ago.

**Figure 8:**
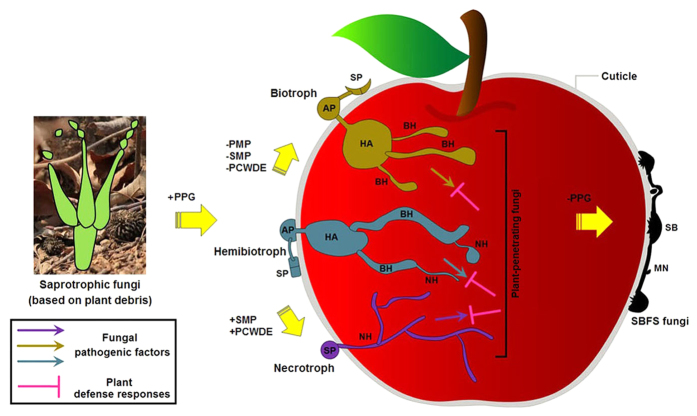
Schematic representation of a hypothesis for the evolutionary route of SBFS fungi. PPG, plant pathogenicity-related gene; PMP, primary metabolism pathway; SMP, secondary metabolism pathway; PCWDE, plant cell wall degrading enzyme; SP, spore; AP, appressorium; HA, haustorium; BH, biotrophic hyphae; NH, necrotrophic hyphae; SB, sclerotium-like body; MN, mycelial network.

In spite of colonizing only the plant cuticle, SBFS pathogens can extract required nutrients from their living hosts without killing cells and tissues. This biotrophic trait, though unconventional (i.e., ectophyte), suffices to place them in the above framework of trophic spectrum and adaptive evolution. Uniquely, SBFS fungi have lost nearly all pathogenicity-related genes except some associated with cuticle penetration and discarded the role of plant cell-penetrating pathogens. Therefore, a separate evolutionary path, i.e., from plant-penetrating parasites to ectophytic SBFS fungi (from interior to exterior colonizers), should be added to the Spanu model^51^ that posited evolution of plant-associated fungi from saprophytism to invasive parasitism (Fig. 8). Driving forces for the evolution from classical plant parasitism to external plant parasitism are unclear, but this change may have facilitated escape from host specialization and thereby enhanced survival during periods of rapid environmental and ecological change.

## Methods

### Fungal strain

*Peltaster fructicola* isolate LNHT1506, selected for both *de novo* genome and transcriptome sequencing, was obtained from a SBFS colony on the surface of a crabapple (*Malus* × *micromalus* Makino) fruit collected in Liaoning Province, China. It is now preserved at −80 °C in the Fungal Herbarium of Northwest A&F University (HMUABO), Yangling, Shaanxi Province, PR China. Prior to experiments, this isolate was re-purified using the single spore isolation method to ensure homozygosity.

### Field inoculation

The *P. fructicola* isolate was used to inoculate apple fruit in the field following the protocol of Batzer *et al.*^9^. To obtain enough inocula, single-conidia-derived colonies on potato dextrose agar (PDA) were cultured for 1 month. After excess agar was removed by scalpel, these colonies were transferred to 1.5-ml sterile plastic centrifuge tubes, 600 μl sterile deionized water (SDW) was added to each of the tubes, and the mixture was shaken on a vortex oscillator (Model QL-901, Kylin-Bell Lab Instruments Company Limited, Haimen City, Jiangsu Province, China) for 60 s. The final inoculum, containing both hyphae and conidia, was acquired by slightly centrifuging the above mixture and then discarding large fragments of thalli at the bottom of tubes.

In an orchard in Yangling, immature fruit (5–6 cm in diameter) of apple (*Malus* × *domestica* ‘Fuji’) from were selected for *in situ* inoculation. Surfaces of these fruit were first sterilized with 70% ethanol using a brush and then allowed to dry for 2 min. In total, 20 fruit were swabbed with inoculum suspension using sterilized brushes, while 10 fruit treated with distilled water served as a control. All 30 fruit were covered *in situ* with polyethylene bags (20 × 15 cm); corners (1.5 × 1.5 cm) of each bag were cut off to facilitate aeration. After 1 to 2 months of incubation, apples with visible colonies of *P. fructicola* were harvested for microscopic examination and total RNA extraction.

### DNA and RNA isolation

Single-spore cultures of *P. fructicola* were grown on PDA for two weeks at 22 °C in darkness before DNA extraction. Highly purified genomic DNA was isolated from the fungal mycelia collected on petri dishes following the modified cetyltrimethyl ammonium bromide (CTAB) protocol^52^. Quality and quantity of total DNA were evaluated using standard 1% agarose gel electrophoresis, as well as spectrophotometrically with NanoDrop 2000 (Thermo Fisher Scientific, USA).

Transcriptome sequencing of *P. fructicola* was conducted using mycelium grown in two different ways: on PDA at 22 °C for 15 days (*in vitro*), and with artificial inoculation onto apple fruit in the field followed by incubation *in situ* (*in vivo*). Total fungal RNA was isolated using a modified hot phenol method^53^. DNA was removed from the extraction with RQ1 DNase (Promega). The quality and quantity of the purified RNA were determined by measuring the absorbance at 260 nm/280 nm (A260/A280) using Smartspec plus (BioRad). RNA integrity was further verified by 1.5% agarose gel electrophoresis.

### Genome sequencing, assembly and analysis

A genomic library of ~500-bp inserts was constructed for the high-throughput solution of *P. fructicola* isolate LNHT1506 and 100-bp paired ends were subsequently sequenced using the Illumina HiSeq2000 platform at the Purdue Genomics Core Facility, West Lafayette, IN, USA. Raw reads from the sequencer were first trimmed by removing the adapters and poor quality bases (score below 20) at both the 5′ and 3′ ends. After adapter removal and quality clipping, any reads below a minimum length (30 bases) were discarded, and the filtered clean reads were finally placed into scaffolds (i.e., actual genome sequence) using the ABySS assembler version 1.3.5^54^. Theoretical size of this genome could be estimated using the raw Illumina sequencing data with JELLYFISH version 2.0^55^. More detailed bioinformatics analyses of the genome are provided in the Supplementary Notes.

### Microscopy of *P. fructicola* on apple fruit surfaces

At harvest maturity, inoculated apples were picked during a 15-day period. Fruit peels displaying colonies of *P. fructicola* were shaved off, cut into 0.5 cm × 0.5 cm segments, fixed with 4% glutaraldehyde in 0.2 mol/L phosphate buffer saline (PBS) (pH 6.8) for 24 h at 4 °C, and washed three times with the same buffer^46^. After dehydration in graded ethanol rinses of 10, 20, 30, 50, 70, 80, 90 and 100% for 15 min, the specimens were dried in a vacuum freeze dryer, mounted on stubs, sputter coated with gold-palladium and observed with a HITACHI scanning electron microscope (S-3400 N) at 5 or 15 kV accelerating voltage.

The epicarps were also cut into 0.3-cm × 0.3-cm segments, fixed with 4% glutaraldehyde in 0.2 mol/L PBS (pH 6.8) for 24 h at 4 °C, washed three times with the same buffer and fixed in 1% osmium tetroxide in 0.1 mol/L PBS (pH 6.8) for 2 h. The samples were then dehydrated by a graded series of ethanol rinses (30, 50, 70, 80, 90 and 100%) and embedded in LR White Resin. Semithin (1.5 μm thick) and ultrathin (80 nm thick) sections were obtained; the former were stained with toluidine blue and observed with a light Olympus BX51 microscope (1000×, oil lens), whereas the latter were stained with uranyl acetate and lead citrate and examined with a HITACHI HT7700 transmission electron microscope at 80 kV.

However, using the above regular methods, we could not observe epicuticular waxes because this layer had probably been dissolved by organic solvents such as ethanol and glutaraldehyde during inoculation and sample preparation. Therefore, to obtain SEM results for the entire cuticle, we first replaced the 70% ethanol (used for surface sterilization) with sterile water, then skipped fixation and dehydration, and directly mounted the specimens (dried naturally for one month) on stubs for sputter coating with gold-palladium^16^.

### cDNA library construction and sequencing

For each treatment, 10 μg of total RNA was used for RNA-seq library construction. Polyadenylated mRNAs were purified and concentrated with oligo(dT)-conjugated magnetic beads (Invitrogen) before being used for directional RNA-seq library construction. Purified mRNAs were iron fragmented at 95 °C followed by end repair and 5′ adaptor ligation. Next, reverse transcription was performed with RT primer harboring 3′ adaptor sequence and randomized hexamer. After cDNAs were purified and amplified, PCR products corresponding to 200–500 bps were purified, quantified and stored at −80 °C until used for sequencing.

During high-throughput sequencing, the libraries were prepared following the manufacturer’s instructions and applied to Illumina NextSeq 500 system for 151 nt pair-end sequencing by ABlife Inc. (Wuhan, China). The sequencing procedure yielded 20,110,886 reads and 7,231,959 reads respectively for *in vitro* and *in vivo* (reads mapped to the apple genome were excluded).

### Transcriptome data processing, mapping and differential expression analysis

Clean data (14,982,373 reads for *in vitro* and 7,231,959 reads for *in vivo*) were acquired by removing the adaptors and the low quality bases (Q < 20) and reads (≥30% of the bases were identified as low quality). After eliminating index sequences and random bases used for balance in library construction, the effective lengths of most clean reads ranged from 145 to 147 nt.

To obtain expression (transcript) profiles for the two treatments, the effective data were mapped separately against the reference *P. fructicola* genome (sequenced in this study) using TopHat2^56^ with up to four read mismatches and one seed mismatch being allowed. Gene expression level for each predicted gene model was quantified by calculating the number of reads per kilobase per million mapped reads (RPKM) in exonic regions^57^. This method makes expression levels of the same genes comparable among different treatments by normalizing the total number of sequencing reads, except for some low-active genes with no reads *in vivo* (because nearly half of the sequencing data belonging to apple are unusable). According to the distribution of RPKM for the gene repositories, high expression (RPKM > 80, approx. 25% of total genes), medium expression (10 < RPKM < 80, approx. 50% of total genes) and low expression (0 < RPKM < 10, approx. 25% of total genes) were designated in this study. Differentially expressed gene (DEG) deep analysis was performed between the above two treatments using the edgeR method^58^ with two judgement parameters (fold change ≥2 or ≤0.5 and *p*-value ≤ 0.01).

## Additional Information

**Accession codes:** This Whole Genome Shotgun project has been deposited at DDBJ/EMBL/GenBank under the accession LJAO00000000 (version LJAO01000000). Raw sequences have been deposited in the NCBI short read archive (SRA) under accession code SRR2188474. The *P. fructicola* genome sequence is available from NCBI under BioProject accession code PRJNA293964 as well as BioSample accession code SAMN04012580.

**How to cite this article**: Xu, C. *et al. Peltaster fructicola* genome reveals evolution from an invasive phytopathogen to an ectophytic parasite. *Sci. Rep.* **6**, 22926; doi: 10.1038/srep22926 (2016).

## Supplementary Material

Supplementary Information
