# Supplementary material for: Peltaster fructicola genome reveals evolution from an invasive phytopathogen to an ectophytic parasite
Source: Sci Rep. 2016 Mar 11;6:22926. doi: 10.1038/srep22926 (PMC4786788; doi:10.1038/srep22926)
Supplement: Supplementary Information [file srep22926-s1.pdf]

Supplementary Information

Peltaster fructicola genome reveals evolution from an invasive phytopathogen to an ectophytic parasite

Chao Xu[1], Huan Chen[1], Mark L. Gleason[2], Jin-Rong Xu[1,3], Huiquan Liu[1], Rong Zhang[1] & Guangyu Sun[1*]

[1]State Key Laboratory of Crop Stress Biology in Arid Areas and College of Plant Protection, Northwest A&F University, Yangling, Shaanxi 712100, China

[2]Department of Plant Pathology and Microbiology, Iowa State University, Ames, Iowa 50011, USA

[3]Department of Botany and Plant Pathology, Purdue University, West Lafayette, Indiana 47907, USA

[*]Corresponding author: e-mail: sgy@nwsuaf.edu.cn; Tel: 086-029-87092075

Supplementary Figures

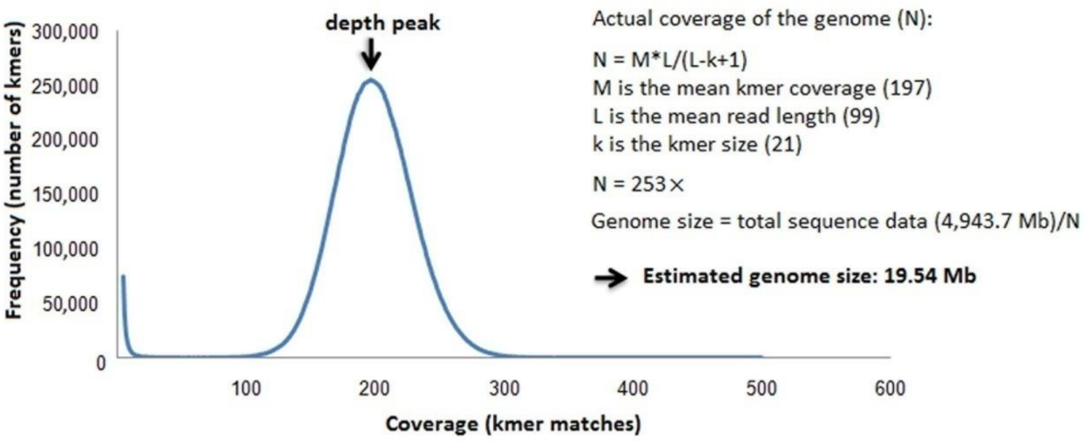

Supplementary Figure 1. Distribution of 21-mer frequency in the raw Illumina genomic reads of *P. fructicola*. The peak depth is at 197X. The peak of 21-mer frequency (M) in reads is correlated with the real sequencing depth (N), read length (L), and kmer length (K); their relationships can be expressed in the experienced formula: M = N * (L – K + 1) / L. We then divided the total sequence length by the real sequencing depth and obtained an estimated genome size of 19.54 Mb.

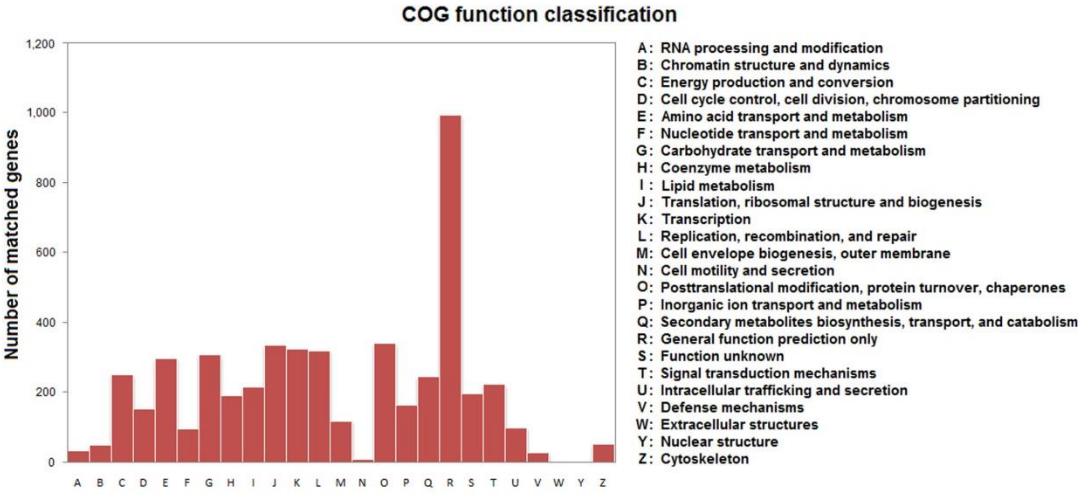

Supplementary Figure S2. Distribution of COG function annotation of the *P. fructicola* genome. In total, there are 3,856 genes (46.3%) that have functional assignments.

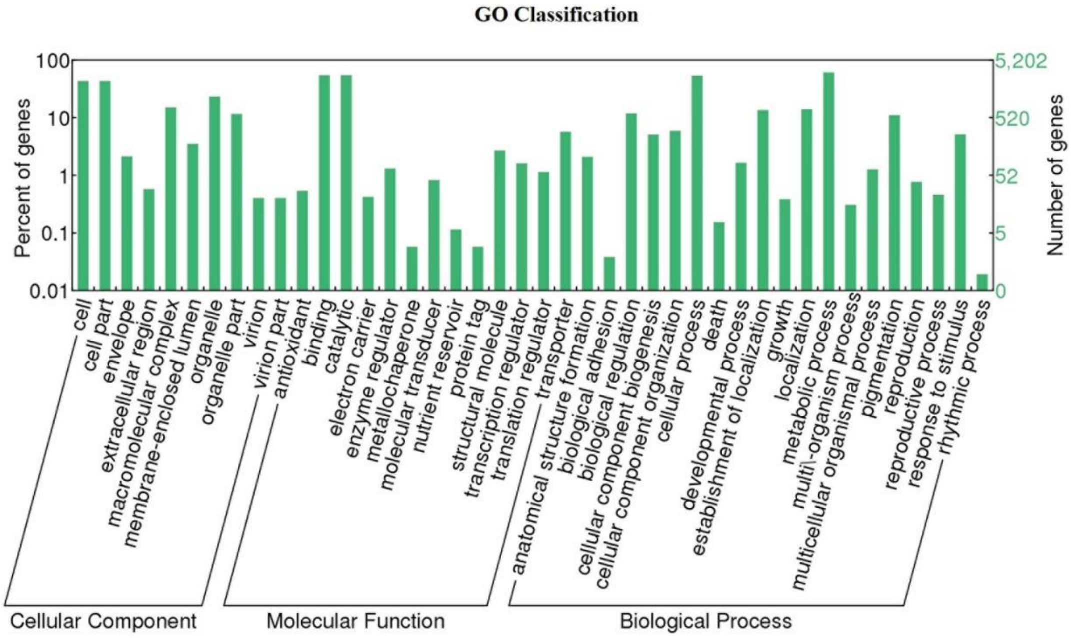

Supplementary Figure S3. GO functional classification of the *P. fructicola* genome. In total, there are 5202 genes (62.4%) that have functional assignments.

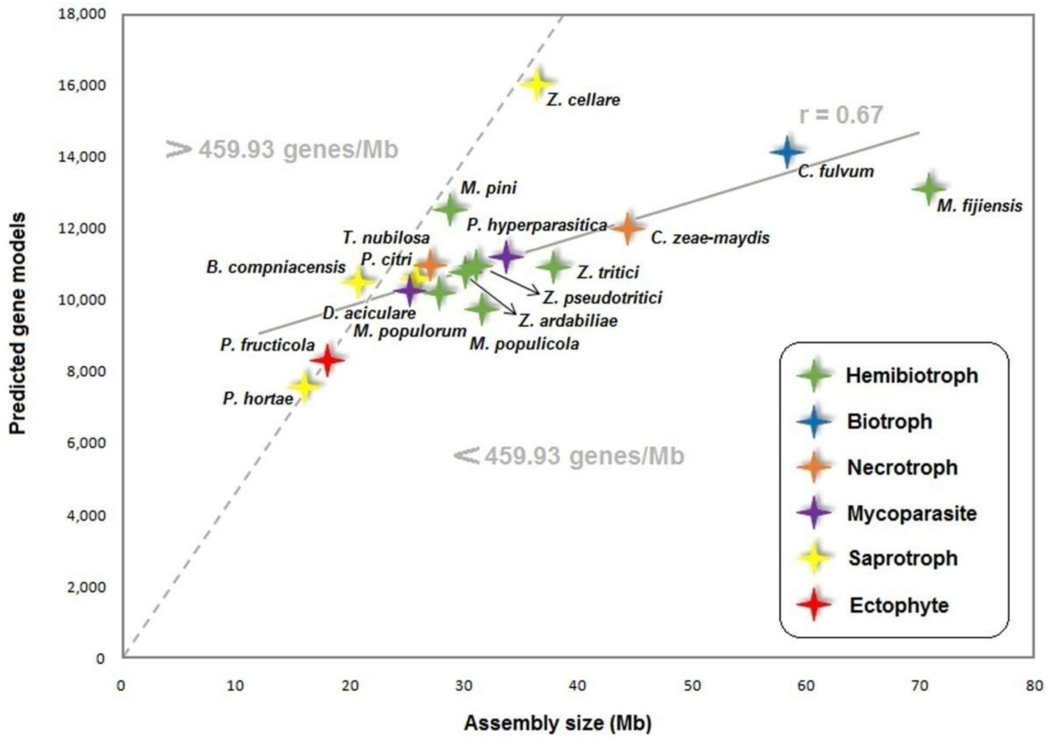

Supplementary Figure S4. Distribution of total gene contents and assembly sizes of sequenced fungi with various lifestyles in Capnodiales. The dashed line discriminates between fungi that have larger and smaller gene densities than *P. fructicola* (459.93 genes/Mb). The solid line indicates the linear trend with the linear correlation coefficient r (0.67). Hemibiotrophs: *Dothistroma septosporum*, *Mycosphaerella fijiensis*, *Zymoseptoria tritici*, *Mycosphaerella populorum*, *Mycosphaerella populicola*, *Zymoseptoria ardabiliae*, *Zymoseptoria pseudotritici*. Biotroph: *Cladosporium fulvum*. Necrotrophs: *Cercospora zeae-maydis*, *Teratosphaeria nubilosa*. Mycoparasites: *Dissoconium aciculare*, *Pseudovirgaria hyperparasitica*. Saprotrophs: *Baudoinia compniacensis*, *Piedraia hortae*, *Polychaeton citri*, *Zasmidium cellare.* Ectophyte: *Peltaster fructicola*.

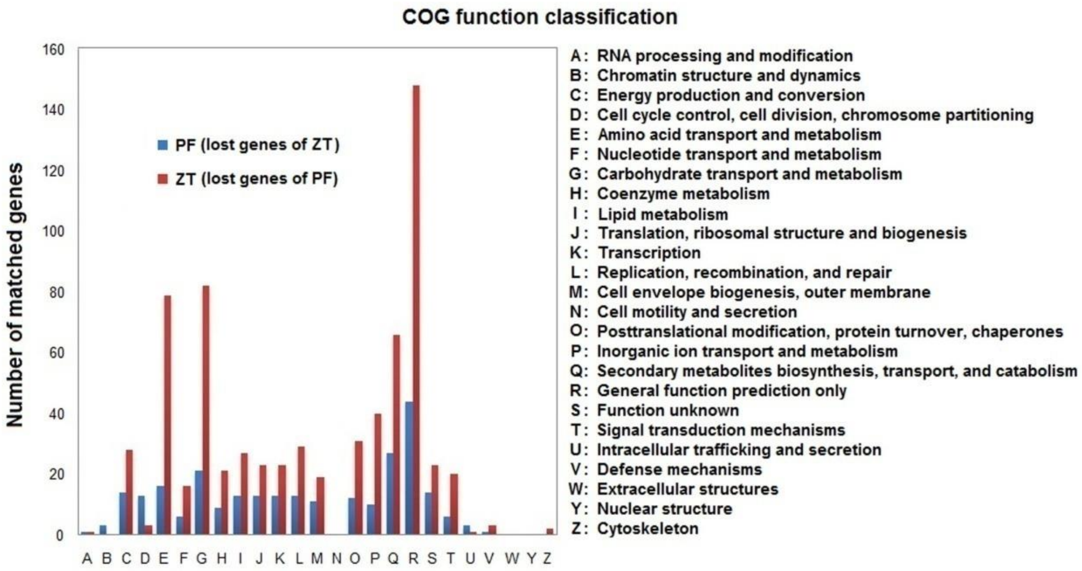

Supplementary Figure S5. Distribution of COG function annotation of the lost genes in *P. fructicola* and *Z. tritici*. In total, 215 genes (38.4%) and 527 genes (45.9%) were annotated in *P. fructicola* (PF) and *Z. tritici* (ZT), respectively.

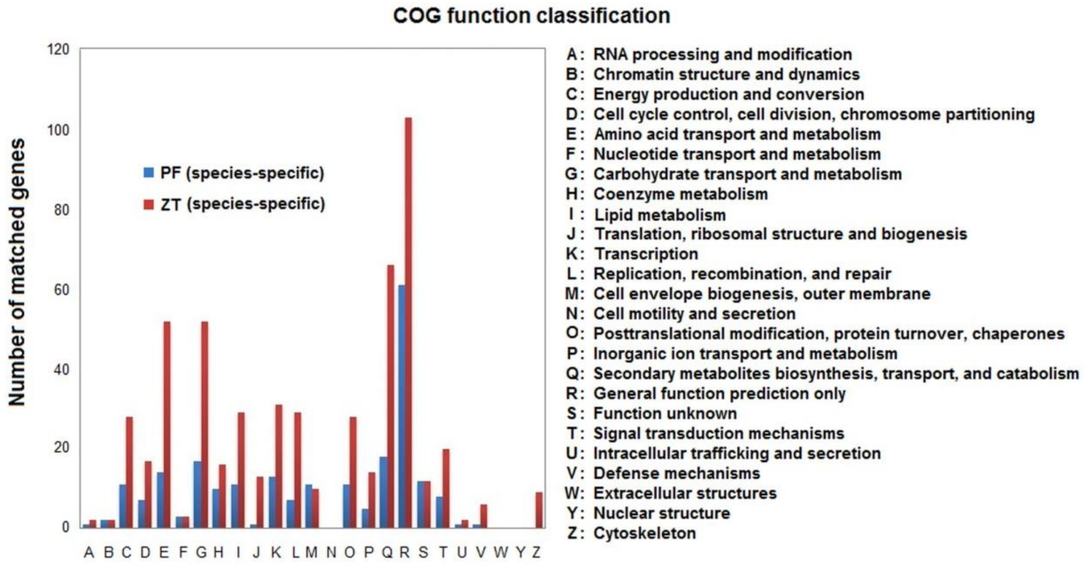

Supplementary Figure S6. Distribution of COG function annotation of species-specific genes in *P. fructicola* and *Z. tritici*. In total, 168 genes (10.3%) and 409 genes (11.7%) were annotated in *P. fructicola* (PF) and *Z. tritici* (ZT), respectively.

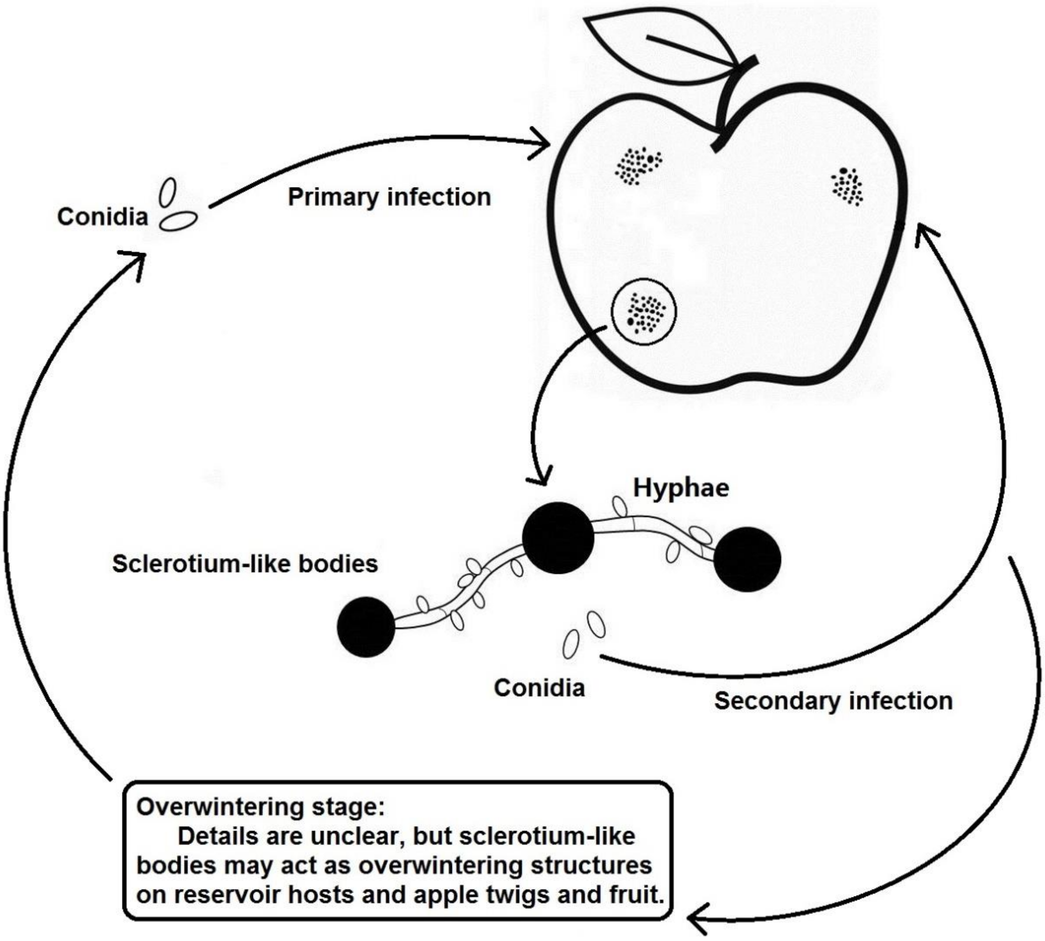

Supplementary Figure S7. The life cycle of *P. fructicola*. In this diagram, only the asexual stage of *P. fructicola* is described because its sexual stage has not been found in either nature or culture.

Supplementary Tables

Supplementary Table S1. Up-regulated and/or highly expressed secreted protein-coding genes *in vivo* in *P. fructicola*.

Gene ID	Pfam annotation	E-value	Nr annotation	E-value	Expression *in vivo*[1]
Pelfr_1250	Glyco_hydro_30 (PF02055)	5.4e-29	Beta-1,6-glucanase	3e-177	Up-regulated & highly expressed
Pelfr_1126	COesterase (PF00135)	6.7e-74	Carboxylesterase type B	0.0	Up-regulated & highly expressed
Pelfr_6771	Metallophos (PF00149)	5.8e-29	Acid phosphatase AphA	0.0	Up-regulated & highly expressed
Pelfr_3889	Metallophos (PF00149)	4.6e-07	Metallo-phosphoesterase	0.0	Up-regulated
Pelfr_4114	Phosphoesterase (PF04185)	9.5e-70	Phospholipase C	0.0	Up-regulated
Pelfr_5583	GDPD (PF03009)	6.6e-07	Glycerophosphoryl diester phosphodiesterase	0.0	Up-regulated
Pelfr_3400	Peroxidase_2 (PF01328)	3.2e-13	Cloroperoxidase	0.0	Up-regulated
Pelfr_2011	FAD_binding_4 (PF01565)	2.5e-25	Secreted FAD-dependent oxidoreductase	4e-96	Up-regulated
Pelfr_5553	Tyrosinase (PF00264)	1.5e-49	Tyrosinase	4e-135	Up-regulated
Pelfr_2833	Arginase (PF00491)	4.2e-83	Arginase/deacetylase	0.0	Up-regulated
Pelfr_4025	Cu-oxidase (PF00394); Cu-oxidase_2 (PF07731); Cu-oxidase_3 (PF07732)	1.1e-44	Multicopper oxidase type 1	0.0	Up-regulated & highly expressed
Pelfr_4790	S1-P1_nuclease (PF02265)	3.2e-96	Nuclease PA3	2e-144	Up-regulated & highly expressed
Pelfr_1369	-	-	Hypothetical protein	4e-57	Up-regulated
Pelfr_2143	DUF1996 (PF09362)	1.2e-59	WSC domain-containing protein	1e-70	Up-regulated
Pelfr_7312	-	-	Hypothetical protein	1e-24	Up-regulated & highly expressed
Pelfr_7724	-	-	Hypothetical protein	1e-10	Up-regulated & highly expressed
Pelfr_3633	Glyco_hydro_17 (PF00332)	6.6e-33	Exo-beta-1,3-glucanase	1e-140	Highly expressed
Pelfr_1460	Peptidase_M28 (PF04389)	1.9e-29	Zinc-metallopeptidase	0.0	Highly expressed
Pelfr_0316	FKBP_C (PF00254)	2.3e-41	FKBP-type peptidyl-prolyl cis-trans isomerase	5e-60	Highly expressed

[1]Up-regulated: expression quantity (RPKM) of in vivo is more than that of in vitro (>=2-fold); Highly expressed: expression quantity (RPKM) of in vivo is more than 80.

Note: in vivo refers to *P. fructicola* infecting apple fruit about 1 month after inoculation; In vitro refers to *P. fructicola* growing on PDA at 22 °C for 15 days; RPKM, reads per kilobase per million mapped reads.

Supplementary Table S2. Secreted peptidases in *P. fructicola*.

Gene ID	Pfam annotation	E-value	Nr annotation	E-value	MEROPS annotation	E-value
Pelfr_5071	Asp (PF00026)	2.6e-33	Aspartic-type endopeptidase	9e-73	PepAc peptidase, subfamily A1A	4.5e-76
Pelfr_3148	Asp (PF00026)	2.0e-52	Aspartic-type endopeptidase	8e-77	PepAc peptidase, subfamily A1A	8.7e-96
Pelfr_4918	Pro-kuma_activ (PF09286)	1.2e-46	Tripeptidyl-peptidase	0.0	Grifolisin, subfamily S53	3.6e-113
Pelfr_1460	Peptidase_M28 (PF04389)	1.9e-29	Zinc-metallopeptidase	0.0	Mername-AA063 peptidase, subfamily M28E	2.6e-110
Pelfr_7574	Peptidase_S8 (PF00082)	1.2e-26	Subtilisin-like protein	5e-37	Subtilisin-like peptidase 3, subfamily S8A	2.3e-26
Pelfr_0800	Peptidase_S10 (PF00450)	3.8e-104	Serine carboxypeptidase	0.0	Carboxypeptidase OcpB, subfamily S10	7.2e-172
Pelfr_6788	Peptidase_S10 (PF00450)	2.03e-82	Serine carboxypeptidase	0.0	Carboxypeptidase O, subfamily S10	1.6e-165

Supplementary Table S3. Small secreted proteins (SSPs) in *P. fructicola*.

Gene ID	Pfam annotation	E-value	Nr annotation	E-value	Cysteine[1]
Pelfr_0039	-	-	Putative carbohydrate-binding protein	7e-54	3.50% (high)
Pelfr_0316	FKBP_C (PF00254)	2.3e-41	FKBP-type peptidyl-prolyl cis-trans isomerase	5e-60	1.46%
Pelfr_1820	His_Phos_1 (PF00300)	1.2e-06	Phosphoglycerate mutase family protein	2e-83	2.87% (high)
Pelfr_3420	-	-	Hypothetical protein	5e-42	7.27% (high)
Pelfr_6255	Ribonuclease (PF00545)	1.3e-18	Ribonuclease	3e-41	2.99% (high)
Pelfr_2097	-	-	Hypothetical protein	4e-25	4.32% (high)
Pelfr_7294	-	-	-	-	1.87%
Pelfr_7319	-	-	-	-	0.00%
Pelfr_7431	-	-	-	-	0.00%
Pelfr_7511	-	-	-	-	0.72%
Pelfr_7794	CVNH (PF08881)	1.3e-07	Cyanovirin-N	9e-07	5.93% (high)
Pelfr_7907	-	-	-	-	4.55% (high)

[1]Values were labeled "high" when the percentage of cysteine residues in the protein was at least twice as high as the average percentage of cysteine residues in all predicted proteins of *P. fructicola*.

Supplementary Table S4. Distribution of putative PCWDEs in selected fungi according to the CAZy database.

Fungi	Total	CE5	CE8	PL1	PL2	PL3	PL4	PL9	PL10	PL11	GH1	GH2	GH3	GH5
Neurospora crassa	87	3	2	1	0	1	1	0	0	0	2	6	10	6
Laccaria bicolor	61	1	4	0	0	0	0	0	0	0	0	2	2	20
Rhizophagus irregularis	12	1	0	0	0	0	0	0	0	0	0	0	0	3
Rhizopus oryzae	61	0	6	0	0	0	0	0	0	0	0	0	6	8
Saccharomyces cerevisiae	18	0	0	0	0	0	0	0	0	0	0	0	0	4
Puccinia graminis	97	9	8	2	0	0	0	0	0	0	0	7	2	28
Ustilago maydis	47	4	1	1	0	0	0	0	0	0	0	1	3	13
Botrytis cinerea	138	12	5	7	0	2	0	0	0	0	4	2	16	16
Colletotrichum higginsianum	226	14	10	16	0	14	6	4	0	0	1	6	22	18
Magnaporthe oryzae	156	19	1	2	0	1	1	0	0	0	2	8	18	13
Zymoseptoria tritici	90	6	1	2	0	1	0	0	0	0	2	8	16	9
Peltaster fructicola	38	6	0	0	0	0	0	0	0	0	1	2	8	7

Fungi	GH6	GH7	GH9	GH10	GH11	GH12	GH26	GH27	GH28	GH29	GH31	GH35	GH36	GH43
Neurospora crassa	3	5	1	4	2	2	1	0	2	0	6	2	0	7
Laccaria bicolor	0	0	1	0	0	3	0	1	6	0	4	1	0	0
Rhizophagus irregularis	0	0	2	0	0	0	1	0	0	0	2	2	0	0
Rhizopus oryzae	0	0	3	0	0	0	1	0	18	0	3	1	1	2
Saccharomyces cerevisiae	0	0	0	0	0	0	0	0	1	0	1	0	0	0
Puccinia graminis	0	10	0	5	0	3	6	7	1	0	2	1	0	2
Ustilago maydis	0	0	1	2	1	0	1	1	1	0	3	1	0	2
Botrytis cinerea	1	3	0	2	2	3	2	4	21	0	0	4	0	6
Colletotrichum higginsianum	3	7	0	7	5	10	1	1	13	1	7	4	1	21
Magnaporthe oryzae	3	7	0	6	5	3	0	3	3	4	6	0	0	20
Zymoseptoria tritici	0	1	1	2	1	1	0	1	2	1	7	2	1	9
Peltaster fructicola	0	0	0	0	0	0	0	1	1	0	5	1	0	1

Fungi	GH45	GH51	GH53	GH54	GH62	GH67	GH74	GH78	GH88	GH93	GH95	GH105	GH115
Neurospora crassa	1	1	1	1	0	1	6	2	0	3	0	2	2
Laccaria bicolor	0	0	0	0	0	0	5	0	1	0	1	9	0
Rhizophagus irregularis	0	0	0	0	0	0	1	0	0	0	0	0	0
Rhizopus oryzae	5	0	0	0	0	0	7	0	0	0	0	0	0
Saccharomyces cerevisiae	0	0	0	0	0	0	12	0	0	0	0	0	0
Puccinia graminis	0	0	0	0	0	0	2	0	0	0	0	2	0
Ustilago maydis	3	2	0	0	1	0	2	0	0	0	0	2	1
Botrytis cinerea	2	2	2	1	1	0	2	7	1	1	3	2	2
Colletotrichum higginsianum	1	2	2	0	1	1	9	5	1	1	1	6	4
Magnaporthe oryzae	1	3	1	1	4	1	6	3	1	1	2	3	4
Zymoseptoria tritici	1	2	2	1	1	0	1	3	0	1	0	3	1
Peltaster fructicola	0	2	0	1	0	0	1	1	0	0	0	0	0

Supplementary Table S5. Manually annotated PCWDE-coding genes in *P. fructicola*.

Gene ID	Substrate	Enzyme activity	Abbreviation	EC number	CAZyme family	Secretion signal
Pelfr_4541	Pectin (backbone chain)	Exopolygalacturonase	PGX	3.2.1.67	GH28	Y
Pelfr_4343	Pectin (backbone/side chain)	α-L-rhamnosidase	RHA	3.2.1.40	GH78	N
Pelfr_0259	Pectin (side chain)/Hemicellulose (Xyloglucan, side chain)	β-1,4-Galactosidase	LAC	3.2.1.23	GH2	N
Pelfr_4976	Pectin (side chain)/Hemicellulose (Xyloglucan, side chain)	β-1,4-Galactosidase	LAC	3.2.1.23	GH35	Y
Pelfr_3609	Pectin (side chain)	Endoarabinase	ABN	3.2.1.99	GH43	N
Pelfr_2065	Pectin (side chain)/Hemicellulose (Xylan, side chain)	α-Arabinofuranosidase	ABF	3.2.1.55	GH51	N
Pelfr_1270	Pectin (side chain)/Hemicellulose (Xylan, side chain)	α-Arabinofuranosidase	ABF	3.2.1.55	GH51	N
Pelfr_5292	Pectin (side chain)/Hemicellulose (Xylan, side chain)	α-Arabinofuranosidase	ABF	3.2.1.55	GH54	N
Pelfr_6616	Pectin (side chain)/Hemicellulose (Xylan, backbone chain)	β-1,4-Xylosidase	BXL	3.2.1.37	GH3	Y
Pelfr_2228	Cellulose (backbone chain)/Hemicellulose (Xyloglucan, backbone chain)	β-1,3-Glucosidase	BGL	3.2.1.21	GH3	N
Pelfr_2067	Cellulose (backbone chain)/Hemicellulose (Xyloglucan, backbone chain)	β-1,4-Glucosidase	BGL	3.2.1.21	GH3	N
Pelfr_1452	Cellulose (backbone chain)/Hemicellulose (Xyloglucan, backbone chain)	β-1,4-Glucosidase	BGL	3.2.1.21	GH3	N
Pelfr_4358	Cellulose (backbone chain)/Hemicellulose (Xyloglucan, backbone chain)	β-1,4-Glucosidase	BGL	3.2.1.21	GH3	N
Pelfr_3458	Cellulose (backbone chain)/Hemicellulose (Xyloglucan, backbone chain)	β-1,4-Glucosidase	BGL	3.2.1.21	GH3	Y
Pelfr_6030	Cellulose (backbone chain)/Hemicellulose (Xyloglucan, backbone chain)	β-1,4-Glucosidase	BGL	3.2.1.21	GH3	Y
Pelfr_6282	Cellulose (backbone chain)/Hemicellulose (Xyloglucan, backbone chain)	β-1,4-Glucosidase	BGL	3.2.1.21	GH1	N
Pelfr_6050	Hemicellulose (Galactomannan, side chain)	α-1,4-Galactosidase	BGL	3.2.1.22	GH27	N
Pelfr_0281	Hemicellulose (Galactomannan, backbone chain)	β-1,4-Mannosidase	MND	3.2.1.25	GH2	Y
Pelfr_2106	Cutin	Cutinase	-	3.1.1.74	CE5	N
Pelfr_1390	Cutin	Cutinase	-	3.1.1.74	CE5	N
Pelfr_5491	Cutin	Cutinase	-	3.1.1.74	CE5	N
Pelfr_3966	Cutin	Cutinase	-	3.1.1.74	CE5	N
Pelfr_3219	Cutin	Cutinase	-	3.1.1.74	CE5	Y
Pelfr_1915	Cutin	Cutinase	-	3.1.1.74	CE5	Y

Supplementary Table S6. Manually annotated PCWDE-coding genes in *Z. tritici*.

Gene ID	Substrate	Enzyme activity	Abbreviation	EC number	CAZyme family	Secretion signal
Mycgr3_66866	Pectin (side chain)	Pectin methyl esterase	PME	3.1.1.11	CE8	Y
Mycgr3_85457	Pectin (backbone chain)	Pectate lyase	PLY	4.2.2.2	PL1	Y
Mycgr3_42327	Pectin (backbone chain)	Pectate lyase	PLY	4.2.2.2	PL1	Y
Mycgr3_27839	Pectin (backbone chain)	Pectate lyase	PLY	4.2.2.2	PL3	N
Mycgr3_86032	Pectin (backbone chain)	Exopolygalacturonase	PGX	3.2.1.67	GH28	N
Mycgr3_77196	Pectin (backbone chain)	Rhamnogalacturonase	RHG	3.2.1.171	GH28	Y
Mycgr3_51381	Pectin (side chain)	β-1,4-Endogalactanase	GAL	3.2.1.89	GH53	N
Mycgr3_25958	Pectin (side chain)	β-1,4-Endogalactanase	GAL	3.2.1.89	GH53	N
Mycgr3_33711	Pectin (backbone/side chain)	α-L-rhamnosidase	RHA	3.2.1.40	GH78	N
Mycgr3_69329	Pectin (backbone/side chain)	α-L-rhamnosidase	RHA	3.2.1.40	GH78	N
Mycgr3_95102	Pectin (side chain)	Exoarabinanase	ABX	3.2.1.-	GH93	Y
Mycgr3_57259	Pectin (backbone chain)	Unsaturated glucuronyl hydrolase	UGH	3.2.1.179	GH105	N
Mycgr3_50077	Pectin (backbone chain)	Unsaturated rhamnogalacturonyl hydrolase	URH	3.2.1.172	GH105	N
Mycgr3_68586	Pectin (side chain)/Hemicellulose (Xyloglucan, side chain)	β-1,4-Galactosidase	LAC	3.2.1.23	GH2	N
Mycgr3_43384	Pectin (side chain)/Hemicellulose (Xyloglucan, side chain)	β-1,4-Galactosidase	LAC	3.2.1.23	GH2	N
Mycgr3_87705	Pectin (side chain)/Hemicellulose (Xyloglucan, side chain)	β-1,4-Galactosidase	LAC	3.2.1.23	GH2	Y
Mycgr3_51250	Pectin (side chain)/Hemicellulose (Xyloglucan, side chain)	β-1,4-Galactosidase	LAC	3.2.1.23	GH2	N
Mycgr3_86272	Pectin (side chain)/Hemicellulose (Xyloglucan, side chain)	β-1,4-Galactosidase	LAC	3.2.1.23	GH2	N
Mycgr3_42647	Pectin (side chain)/Hemicellulose (Xyloglucan, side chain)	β-1,4-Galactosidase	LAC	3.2.1.23	GH2	N
Mycgr3_75961	Pectin (side chain)/Hemicellulose (Xyloglucan, side chain)	β-1,4-Galactosidase	LAC	3.2.1.23	GH35	N
Mycgr3_101693	Pectin (side chain)/Hemicellulose	β-1,4-Galactosidase	LAC	3.2.1.23	GH35	N

	(Xyloglucan, side chain)					
Mycgr3_105728	Pectin (side chain)/Hemicellulose (Xylan, backbone chain)	β-1,4-Xylosidase	BXL	3.2.1.37	GH43	Y
Mycgr3_75584	Pectin (side chain)/Hemicellulose (Xylan, backbone chain)	β-1,4-Xylosidase	BXL	3.2.1.37	GH43	Y
Mycgr3_73095	Pectin (side chain)/Hemicellulose (Xylan, backbone chain)	β-1,4-Xylosidase	BXL	3.2.1.37	GH43	N
Mycgr3_105323	Pectin (side chain)	Endoarabinase	ABN	3.2.1.99	GH43	Y
Mycgr3_30121	Pectin (side chain)/Hemicellulose (Xylan, backbone chain)	β-1,4-Xylosidase	BXL	3.2.1.37	GH43	Y
Mycgr3_98714	Pectin (side chain)/Hemicellulose (Xylan, backbone chain)	β-1,4-Xylosidase	BXL	3.2.1.37	GH43	N
Mycgr3_84076	Pectin (side chain)/Hemicellulose (Xylan, backbone chain)	β-1,4-Xylosidase	BXL	3.2.1.37	GH43	N
Mycgr3_96505	Pectin (side chain)/Hemicellulose (Xylan, backbone chain)	β-1,4-Xylosidase	BXL	3.2.1.37	GH43	Y
Mycgr3_40215	Pectin (side chain)/Hemicellulose (Xylan, side chain)	α-Arabinofuranosidase	ABF	3.2.1.55	GH51	N
Mycgr3_111130	Pectin (side chain)/Hemicellulose (Xylan, side chain)	α-Arabinofuranosidase	ABF	3.2.1.55	GH51	Y
Mycgr3_70396	Pectin (side chain)/Hemicellulose (Xylan, side chain)	α-Arabinofuranosidase	ABF	3.2.1.55	GH54	Y
Mycgr3_68922	Pectin (side chain)/Hemicellulose (Xylan, side chain)	α-Arabinofuranosidase	ABF	3.2.1.55	GH62	Y
Mycgr3_71284	Pectin (side chain)/Hemicellulose (Xylan, backbone chain)	β-1,4-Xylosidase	BXL	3.2.1.37	GH3	Y
Mycgr3_38178	Pectin (side chain)/Hemicellulose (Xylan, backbone chain)	β-1,4-Xylosidase	BXL	3.2.1.37	GH3	N
Mycgr3_85505	Cellulose (backbone chain)/Hemicellulose (Xyloglucan, backbone	β-1,4-Glucosidase	BGL	3.2.1.21	GH3	Y

	chain)					
Mycgr3_41307	Cellulose (backbone chain)/Hemicellulose (Xyloglucan, backbone chain)	β-1,4-Glucosidase	BGL	3.2.1.21	GH3	N
Mycgr3_99970	Cellulose (backbone chain)/Hemicellulose (Xyloglucan, backbone chain)	β-1,4-Glucosidase	BGL	3.2.1.21	GH3	Y
Mycgr3_45540	Cellulose (backbone chain)/Hemicellulose (Xyloglucan, backbone chain)	β-1,4-Glucosidase	BGL	3.2.1.21	GH3	N
Mycgr3_107206	Cellulose (backbone chain)/Hemicellulose (Xyloglucan, backbone chain)	β-1,4-Glucosidase	BGL	3.2.1.21	GH3	N
Mycgr3_20995	Cellulose (backbone chain)/Hemicellulose (Xyloglucan, backbone chain)	β-1,4-Glucosidase	BGL	3.2.1.21	GH3	N
Mycgr3_54587	Cellulose (backbone chain)/Hemicellulose (Xyloglucan, backbone chain)	β-1,4-Glucosidase	BGL	3.2.1.21	GH3	N
Mycgr3_38222	Cellulose (backbone chain)/Hemicellulose (Xyloglucan, backbone chain)	β-1,4-Glucosidase	BGL	3.2.1.21	GH3	N
Mycgr3_42620	Cellulose (backbone chain)/Hemicellulose (Xyloglucan, backbone chain)	β-1,4-Glucosidase	BGL	3.2.1.21	GH3	N
Mycgr3_42323	Cellulose (backbone chain)/Hemicellulose (Xyloglucan, backbone chain)	β-1,4-Glucosidase	BGL	3.2.1.21	GH3	Y
Mycgr3_64142	Cellulose (backbone chain)/Hemicellulose (Xyloglucan, backbone chain)	β-1,4-Glucosidase	BGL	3.2.1.21	GH3	Y
Mycgr3_77647	Cellulose (backbone chain)/Hemicellulose (Xyloglucan, backbone	β-1,4-Glucosidase	BGL	3.2.1.21	GH3	N

	chain)					
Mycgr3_70973	Cellulose (backbone chain)/Hemicellulose (Xyloglucan, backbone chain)	β-1,4-Glucosidase	BGL	3.2.1.21	GH1	N
Mycgr3_49899	Cellulose (backbone chain)/Hemicellulose (Xyloglucan, backbone chain)	β-1,4-Glucosidase	BGL	3.2.1.21	GH1	Y
Mycgr3_100252	Cellulose (backbone chain)/Hemicellulose (Xyloglucan, backbone chain)	Cellobiohydrolase I	CBHI	3.2.1.176	GH7	N
Mycgr3_76589	Cellulose (backbone chain)/Hemicellulose (Xyloglucan, backbone chain)	β-1,4-Endoglucanase	EG	3.2.1.4	GH45	Y
Mycgr3_71664	Cellulose (backbone chain)/Hemicellulose (Xyloglucan, backbone chain)	β-1,4-Endoglucanase	EG	3.2.1.4	GH5	N
Mycgr3_105871	Cellulose (backbone chain)/Hemicellulose (Xyloglucan, backbone chain)	β-1,4-Endoglucanase	EG	3.2.1.4	GH12	Y
Mycgr3_94846	Hemicellulose (Xylan, backbone chain)	β-1,4-Endoxylanase	XLN	3.2.1.8	GH10	Y
Mycgr3_61141	Hemicellulose (Xylan, backbone chain)	β-1,4-Endoxylanase	XLN	3.2.1.8	GH10	Y
Mycgr3_60105	Hemicellulose (Xylan, backbone chain)	β-1,4-Endoxylanase	XLN	3.2.1.8	GH11	Y
Mycgr3_107208	Hemicellulose (Galactomannan, side chain)	α-1,4-Galactosidase	AGL	3.2.1.22	GH27	N
Mycgr3_110289	Hemicellulose (Galactomannan, side chain)	α-1,4-Galactosidase	AGL	3.2.1.22	GH36	Y
Mycgr3_68054	Hemicellulose (Galactomannan, backbone chain)	β-1,4-Mannosidase	MND	3.2.1.25	GH2	N
Mycgr3_107565	Hemicellulose (Galactomannan, backbone chain)	β-1,4-Mannosidase	MND	3.2.1.25	GH2	Y
Mycgr3_92097	Hemicellulose	β-1,4-Endomannanase	MAN	3.2.1.78	GH5	Y

	(Galactomannan, backbone chain)					
Mycgr3_38237	Hemicellulose (Xyloglucan, side chain)	α-Fucosidase	AFC	3.2.1.51	GH29	N
Mycgr3_68483	Cutin	Cutinase	-	3.1.1.74	CE5	Y
Mycgr3_99331	Cutin	Cutinase	-	3.1.1.74	CE5	Y
Mycgr3_18212	Cutin	Cutinase	-	3.1.1.74	CE5	N
Mycgr3_43394	Cutin	Cutinase	-	3.1.1.74	CE5	Y
Mycgr3_35055	Cutin	Cutinase	-	3.1.1.74	CE5	N
Mycgr3_77282	Cutin	Cutinase	-	3.1.1.74	CE5	Y

Supplementary Table S7. Secondary metabolism (SM) key genes in *P. fructicola*.

Gene ID	SM cluster	SM class	Nr annotation	E-value
Pelfr_2368	Cluster1	PKS	Conidial pigment biosynthesis polyketide synthase	0.0
Pelfr_4242	Cluster2	PKS	Conidial yellow pigment biosynthesis polyketide synthase	0.0
Pelfr_6860	Cluster3	PKS	Lovastatin nonaketide synthase	0.0
Pelfr_1160	Cluster4	PKS-NRPS	Hybrid PKS-NRPS enzyme	0.0
Pelfr_0875	Cluster5	PKS-NRPS	Lovastatin nonaketide synthase	0.0
Pelfr_0462	Cluster6	NRPS	HC-toxin synthetase-like protein	1e-151
Pelfr_0485	Cluster6	NRPS	HC-toxin synthetase-like protein	0.0
Pelfr_0978	Cluster7	NRPS	Linear gramicidin synthase subunit D	0.0
Pelfr_6327	Cluster8	NRPS	Nonribosomal siderophore peptide synthase	0.0
Pelfr_6213	Cluster9	NRPS	Linear gramicidin synthetase subunit C	0.0
Pelfr_6610	Cluster10	NRPS	Peroxisomal-coenzyme A synthetase	0.0
Pelfr_3849	Cluster11	NRPS	Acetyl-CoA synthetase-like protein	0.0
Pelfr_1264	Cluster12	TS	Squalene synthase	0.0
Pelfr_6032	Cluster13	TS	Geranylgeranyl diphosphate synthase	0.0
Pelfr_4219	Cluster14	TS	Lycopene beta-cyclase	0.0

Supplementary Table S8. DHN-melanin biothythesis-related genes in *P. fructicola*.

Gene ID	Gene name	Description	GenBank number of reference gene[1]	Identity	Similarity
Pelfr_2368	PKS18	Polyketide synthase	AY495659	65%	76%
Pelfr_6774	BRN2	T4HN reductase	EF060260	82%	91%
Pelfr_0341	SCD1	Scytalone dehydratase	EF060261	69%	83%
Pelfr_2393	BRN1	T3HN reductase	AB001564	78%	87%

[1]GenBank numbers of the referenced gene models are from *Cochliobolus heterostrophus*.

Supplementary Table S9. Genes and their expression, which involve in four major signal transduction pathways, autophagy and Ros in *P. fructicola* and related species.

Gene name	*M. oryzae*	*Z. tritici*	*P. fructicola*	*In vivo*[1]	*In vitro*[2]
**cAMP-PKA pathway: pathogenicity, surface recognition, and turgor generation**					
Cap1	MGG_01722	Mycgr3_76018	Pelfr_1322	77.62	51.78
Cpk2	MGG_02832	Mycgr3_38697	Pelfr_6488	0	24.24
CpkA	MGG_06368	Mycgr3_99725	Pelfr_2492	5.07	52.35
Ctdf	MGG_11346	Mycgr3_89219	Pelfr_8173	0	0
Mac1	MGG_09898	Mycgr3_86659	Pelfr_5544	8.45	52.81
MagA	MGG_01818	Mycgr3_76503	Pelfr_1576	12.05	191.43
MagB	MGG_00365	Mycgr3_52873	Pelfr_5694	0.53	65.31
Mgb1	MGG_05201	Mycgr3_68892	Pelfr_6673	0	13.2
Mgg1	MGG_10193	Mycgr3_102649	Pelfr_0632	20.11	201.07
Mstu1	MGG_00692	Mycgr3_93828	Pelfr_4840	4.19	60.81
PdeH	MGG_05664	Mycgr3_33550	Pelfr_0043	18.16	37.6
Pth11	MGG_05871	Mycgr3_16078	Pelfr_1389	12.48	48.99
Pth12	MGG_12865	-	-	-	-
Rgs1	MGG_14517	Mycgr3_50182	Pelfr_1219	0	11.19
Som1	MGG_04708	Mycgr3_109061	Pelfr_0213	11.44	31.45
**Pmk1 pathway: appressorium formation, pathogenicity, and invasive growth**					
Cdc42	MGG_00466	Mycgr3_99553	Pelfr_2379	0	155.43
Gap1	MGG_11062	Mycgr3_86730	Pelfr_1799	227.76	206.74
Gas1	MGG_12337	-	-	-	-
Gas2	MGG_04202	-	-	-	-
Mcm1	MGG_02773	Mycgr3_31170	Pelfr_2334	24.51	122.36
Msb2	MGG_06033	-	Pelfr_2676	53.06	247.9
Mst7	MGG_00800	Mycgr3_52389	Pelfr_2369	0.41	104.79
Mst11	MGG_14847	Mycgr3_71128	Pelfr_0435	54.77	49.18
Mst12	MGG_12958	Mycgr3_46840	Pelfr_4757	12.95	212.64
Mst50	MGG_05199	Mycgr3_91070	Pelfr_6694	15.19	55.78
Pic5	MGG_08600	Mycgr3_100168	Pelfr_0651	37.83	19.23
Pmk1	MGG_09565	Mycgr3_73071	Pelfr_4994	0	96.23
Ras1	MGG_06154	Mycgr3_75299	Pelfr_4161	64.57	246.68
Ras2	MGG_09499	Mycgr3_59862	Pelfr_5632	0	58.67
Rgf1	MGG_03064	Mycgr3_57099	Pelfr_6633	99.02	59.32
Sfl1	MGG_06971	Mycgr3_38379	Pelfr_6316	18.59	42.51
Sho1	MGG_09125	Mycgr3_108657	Pelfr_6493	45.94	191.34
**Mps1 pathway: pathogenicity, penetration, cell wall integrity, and conidiation**					
Cch1	MGG_05643	Mycgr3_32076	Pelfr_3280	103.55	51.04
Cna1	MGG_07456	Mycgr3_100967	Pelfr_3472	28.61	90.75
Cnb1	MGG_06933	Mycgr3_68731	Pelfr_5246	13.52	260.24
Crz1	MGG_05133	Mycgr3_117712	Pelfr_1135	20.69	43.23
Mck1	MGG_00883	-	Pelfr_2301	3.48	18.81
Mig1	MGG_01204	Mycgr3_117729	Pelfr_5664	2.89	36.74

Mkk1	MGG_06482	Mycgr3_71299	Pelfr_6025	9.15	213.35
Mkk2	MGG_00800	Mycgr3_52389	Pelfr_2369	0.41	104.79
Mps1	MGG_04943	Mycgr3_102121	Pelfr_1869	53.27	287.2
Pkc1	MGG_08689	Mycgr3_87605	Pelfr_4305	12.31	47.68
Plc1	MGG_02444	Mycgr3_66273	Pelfr_5462	0	14.59
Pmc1	MGG_02487	Mycgr3_35226	Pelfr_3000	19.93	55.7
Rho1	MGG_07176	Mycgr3_106397	Pelfr_0710	164.12	892.02
Swi6	MGG_09869	Mycgr3_48696	Pelfr_1581	2.82	16.89
**Oms1 pathway: osmoregulation, stress response, and fungicide sensitivity**					
Ap1	MGG_12814	Mycgr3_35076	Pelfr_3144	83.7	89.36
Atf1	MGG_08212	Mycgr3_98304	Pelfr_6217	45.26	127.81
Hik1	MGG_11174	Mycgr3_35302	Pelfr_0583	86	67.86
Oms1	MGG_01822	Mycgr3_76502	Pelfr_1558	1.87	534
Pbs2	MGG_10268	Mycgr3_76249	Pelfr_5316	65.72	121.39
Skn7	MGG_03516	Mycgr3_109990	Pelfr_3787	14.4	41.27
Sln1	MGG_07312	Mycgr3_63909	Pelfr_0738	18.23	47.59
SSk1	MGG_02897	Mycgr3_70181	Pelfr_5884	5.1	108.13
Ssk2	MGG_00183	Mycgr3_67344	Pelfr_3555	5.52	22.77
Ypd1	MGG_07173	Mycgr3_32785	Pelfr_0942	23.6	184.13
**Autophagy-related genes: conidiation, appressorium formation and pathogenicity**					
Atg1	MGG_06393	Mycgr3_73716	Pelfr_1850	0.83	16.61
Atg2	MGG_05998	Mycgr3_83423	Pelfr_1077	1.14	11.44
Atg3	MGG_02959	Mycgr3_99699	Pelfr_1591	13.75	173.12
Atg4	MGG_03580	Mycgr3_49421	Pelfr_1738	0	58.2
Atg5	MGG_09262	Mycgr3_86699	Pelfr_2519	0	1.28
Atg6	MGG_03694	Mycgr3_72630	Pelfr_6195	3.26	51.55
Atg7	MGG_07297	Mycgr3_100434	Pelfr_4877	43.06	55.44
Atg8	MGG_01062	-	Pelfr_3311	190.88	1511.44
Atg9	MGG_09559	Mycgr3_100550	Pelfr_3729	14.7	75.6
Atg10	MGG_14737	-	Pelfr_1301	0	7.06
Atg12	MGG_00598	Mycgr3_40907	Pelfr_3199	0	7.09
Atg13	MGG_00454	Mycgr3_58223	Pelfr_3154	2.6	44.51
Atg15	MGG_12828	Mycgr3_108706	Pelfr_6428	11	68.77
Atg16	MGG_05255	Mycgr3_90647	Pelfr_6215	0	156.39
Atg17	MGG_07667	Mycgr3_70424	Pelfr_6704	21.13	18.96
Atg18	MGG_03139	Mycgr3_75475	Pelfr_5073	9.29	61.54
**Ros related genes: conidium germination and appressorium formation**					
Des1	MGG_04163	-	-	-	-
Hyr1	MGG_07460	Mycgr3_102589	Pelfr_5909	161.59	535.79
Nox1	MGG_00750	Mycgr3_65626	Pelfr_0062	0	32.21
Nox2	MGG_06559	-	-	-	-
NoxR	MGG_05280	Mycgr3_67898	Pelfr_6118	32.13	120.48
Rac1	MGG_02731	Mycgr3_106564	Pelfr_4271	1.51	164.27

Rho3	MGG_10323	Mycgr3_64768	Pelfr_4285	14.82	127.61

[1]Gene expression was quantified by RPKM in RNA-seq analysis; RPKM, reads per kilobase per million mapped reads.

[2]*In vivo* refers to transcripts expressed by *P. fructicola* infecting apple fruit about 1 month after inoculation; *In vitro* refers to transcripts expressed by *P. fructicola* growing on PDA at 22 °C for 15 days.

Note: Orthologous genes were aligned in the same lines.

Supplementary Table S10. Fungi used for identification of multigene families and phylogenetic analysis in this study.

Species	Taxonomy	Lifestyle	Gene number
Laccaria bicolor	Basidiomycota	Symbiosis	23,132
Rhizophagus irregularis	Glomeromycota	Symbiosis	30,282
Rhizopus oryzae	Zygomycota	Saprotroph	17,467
Saccharomyces cerevisiae	Ascomycota	Saprotroph	6,575
Neurospora crassa	Ascomycota	Saprotroph	10,785
Puccinia graminis	Basidiomycota	Biotroph	20,534
Ustilago maydis	Basidiomycota	Biotroph	6,522
Botrytis cinerea	Ascomycota	Necrotroph	16,447
Colletotrichum higginsianum	Ascomycota	Hemibiotroph	16,172
Magnaporthe oryzae	Ascomycota	Hemibiotroph	11,054
Zymoseptoria tritici	Ascomycota	Hemibiotroph	10,933
Peltaster fructicola	Ascomycota	Ectophyte	8,334

Supplementary Table S11. Summary of CAZyme families involved in plant cell wall degradation.

Substrate		Enzyme activity	Abbreviation	EC number	CAZyme family(ies)
Cutin	Backbone chain	Cutinase	-	3.1.1.74	CE5
Cellulose	Backbone chain	β-1,4-Endoglucanase	EG	3.2.1.4	GH3, -5, -6, -7, -9, -12, -45
	Backbone chain	Cellobiohydrolase (reducing end)	CBHI	3.2.1.176	GH7
	Backbone chain	Cellobiohydrolase (nonreducing end)	CBHII	3.2.1.91	GH6
	Backbone chain	β-1,4-Glucosidase	BGL	3.2.1.21	GH1, -3
Pectin	Backbone chain	Endopolygalacturonase	PGA	3.2.1.15	GH28
	Backbone chain	Exopolygalacturonase	PGX	3.2.1.67	GH28
	Backbone chain	Endo-xylogalacturonan hydrolase	XGH	3.2.1.-	GH28
	Backbone chain	Rhamnogalacturonase	RHG	3.2.1.171	GH28
	Backbone chain	Rhamnogalacturonan α-1,2-galacturonohydrolase	RHX	3.2.1.173	GH28
	Backbone chain	Rhamnogalacturonan α-L-rhamnopyranohydrolase	RGXB	3.2.1.174	GH28
	Backbone/side chain	α-L-rhamnosidase	RHA	3.2.1.40	GH78
	Backbone chain	Pectin lyase	PEL	4.2.2.10	PL1
	Backbone chain	Pectate lyase	PLY	4.2.2.2	PL1, -2, -3, -9, -10
	Backbone chain	Rhamnogalacturonan lyase	RGL	4.2.2.23	PL4, -11
	Backbone chain	Unsaturated rhamnogalacturonan hydrolase	URH (*Bacillus subtilis*)	3.2.1.172	GH105
	Backbone chain	Unsaturated glucuronyl hydrolase	UGH (*Bacillus sp.*)	3.2.1.179	GH88, -105
	Side chain	Endoarabinanase	ABN	3.2.1.99	GH43
	Side chain	Exoarabinanase	ABX	3.2.1.-	GH93
	Side chain	β-1,4-Endogalactanase	GAL	3.2.1.89	GH53
	Side chain	α-Arabinofuranosidase	ABF	3.2.1.55	GH51, -54, -62
	Side chain	β-1,4-Xylosidase	BXL	3.2.1.37	GH3, -43
	Side chain	β-1,4-Galactosidase	LAC	3.2.1.23	GH2, -35
	Side chain	Pectin methyl esterase	PME	3.1.1.11	CE8
Xylan/Heteroxylan	Backbone chain	β-1,4-Endoxylanase	XLN	3.2.1.8	GH10, -11
	Backbone chain	β-1,4-Xylosidase	BXL	3.2.1.37	GH3, -43
	Side chain	α-Arabinofuranosidase	ABF	3.2.1.55	GH51, -54, -62
	Side chain	α-Glucuronidase	AGU	3.2.1.139	GH67, -115
Galactomannan	Backbone chain	β-1,4-Endomannanase	MAN	3.2.1.78	GH5, -26
	Backbone chain	β-1,4-Mannosidase	MND	3.2.1.25	GH2
	Side chain	α-1,4-Galactosidase	AGL	3.2.1.22	GH27, -36

Xyloglucan	Backbone chain	Xyloglucan β-1,4-endoglucanase	XEG	3.2.1.151	GH12, -74
	Backbone chain	β-1,4-Endoglucanase	EG	3.2.1.4	GH3, -5, -6, -7, -9, -12, -45
	Backbone chain	Cellobiohydrolase (reducing end)	CBHI	3.2.1.176	GH7
	Backbone chain	Cellobiohydrolase (nonreducing end)	CBHII	3.2.1.91	GH6
	Backbone chain	β-1,4-Glucosidase	BGL	3.2.1.21	GH1, -3
	Side chain	α-Xylosidase	AXL	3.2.1.177	GH31
	Side chain	α-Fucosidase	AFC	3.2.1.51	GH29, -95
	Side chain	β-1,4-Galactosidase	LAC	3.2.1.23	GH2, -35

Supplementary Notes

Gene prediction and annotation.

The draft genome of *P. fructicola* was closed by GapFiller[1] with the dataset of paired reads, then masked using RepeatMasker[2] with both the latest RepBase fungal library[3] and *ab initio* library generated by RepeatModeler[2]. The modified assembly containing only scaffolds larger than 200 bp was used as input for gene model prediction and other downstream analyses. Gene calling was conducted using an annotation pipeline and genome-database management tool MAKER2[4], which combines three different *ab initio* predictors: GeneMark-ES[5], Augustus[6], and SNAP[7]. In addition, proteomes of several fungi within the same order as *P. fructicola* were downloaded from JGI Fungal Genome Portal MycoCosm[8] and served as experimental evidence for quality control of the result of the above computational methods. A set of 248 CEGs identified by Parra *et al.*[9] were used to evaluate the completeness of the gene space in the genome of *P. fructicola*.

All predicted gene models were functionally annotated using an all-in-one bioinformatics software suite, Blast2GO, based on the Gene Ontology vocabulary[10]. This high-throughput functional annotation pipeline integrates various annotation strategies and tools, including BLASTp alignment against GenBank with non-redundant set[11], GO (Gene Ontology) terms mapping[12], InterProScan[13], Enzyme Commission (EC) numbers assigning[14], and Kyoto Encyclopedia of Genes and Genomes (KEGG)[15]. In addition, they were subjected to Conserved Domains Database (CDD)[16], Pfam[17], and Clusters of Orthologous Group (COG) analysis[18]. The rRNA-coding region (rDNA) was designated using RNAmmer[19], but the copy number of rDNA (tandem repeat and low polymorphism)[20] in the assembly could be estimated only by calculating its average sequencing depth (divided by the global depth).

Multigene families and phylogenetic analysis.

Eleven representative fungi (Supplementary Table S10) that collectively span diverse lifestyles (symbiotic, saprophytic, biotrophic, hemibiotrophic and necrotrophic) and phyla (Ascomycota, Basidiomycota, Zygomycota and Glomeromycota) were selected to predict multigene families along with *P. fructicola*. Gene clusters containing both orthologs and paralogs were built among a total of 178,237 proteins (retrieved from JGI Fungal Genome Portal MycoCosm except *P. fructicola*) using OrthoMCL[21] based on the Markov cluster algorithm (MCL)[22].

To construct a genome-based phylogenetic tree of the above 12 species, single-copy orthologous families (having exactly one gene for each organism) were collected from the MCL clustering result. Multiple alignments of the protein sequences belonging to the same families were parallelly performed in batch mode using MAFFT[23]. Conservative positions in the alignment result were extracted through GBlocks[24] and then used to estimate the best protein evolution model with ProtTest 3.4[25]. Maximum-likelihood phylogenetic analysis of the concatenated well-aligned regions was carried out using RAxML[26] with a bootstrap value of 1000 replications. The RAxML tree obtained was visualized in FigTree v1.4.2 (http://tree.bio.ed.ac.uk/software/). The divergence times between species were estimated with the method provided by MEGA 6[27], using the previously published calibration points as a reference: Ascomycota-Basidiomycota split 500 Myr, and Pezizomycotina-Saccharomycotina split 350 Myr[28].

We also constructed the phylogenetic profiles for all gene families to reflect the absence or presence of a particular gene family in a given species. These profiles were combined with the

above species tree using the DOLLOP program from the PHYLIP package[29] to reconstruct the parsimonious series of gene gain and loss events for these fungal genomes. To define the minimum gene set for each ancestral node, the DOLLOP program was used, which follows the Dollo parsimony principle assuming that every gene appears exactly once on the tree and can be lost independently in different evolutionary lineages.

CAZymes and plant cell wall degrading enzymes.

Proteomes were screened for carbohydrate-active modules using a web server for automated carbohydrate-active enzyme (CAZyme) annotation, dbCAN[30]. CAZyme module annotation by this program uses E-value, alignment length and coverage, with an E-value of <1e-5 for alignments of >80 amino acids and an E-value of <1e-3 for alignments of <80 amino acids. To eliminate those selected by dbCAN but not really CAZymes, proteins with CAZyme motifs with a dbCAN E-value >e-10 were individually examined using two online tools, Pfam[17] and BLASTp of non-redundant protein sequences in NCBI (http://www.ncbi.nlm.nih.gov).

Cellulases, hemicellulases, pectinases and cutinases collectively constituted the plant cell wall-degrading enzymes (PCWDEs) that we considered in this study. According to previous research[31-33], the above four types of PCWDEs are distributed in a range of different CAZyme families (despite including some non-PCWDEs) (Supplementary Table S11) belonging to three functional classes, i.e., glycoside hydrolases (GHs), polysaccharide lyases (PLs), and carbohydrate esterases (CEs). We could therefore pick out these PCWDE-involved families from the predicted CAZymes and considered them as putative PCWDEs. Additionally, to identify the function of each putative PCWDE more accurately and discard the false positives, the BLASTp program and two online databases, mycoCLAP[34] and Fungal PCWDE Database[35] were jointly applied.

Secretomes, secreted peptidases and small secreted proteins.

In the current study, secretomes were identified using a custom pipeline including SignalP version 4.1[36], TMHMM Server version 2.0[37], TargetP 1.1 Server[38], GPIsom[39] and WoLF PSORT[40]. Initially, all proteins of one organism with a SignalP SP = 'YES' or a TargetP Loc = S were selected and combined[41]. These proteins were then scanned for transmembrane (TM) domains using TMHMM and those with 0 TM or 1 TM, if located in or very close to the predicted N-terminal signal peptide (this often corresponds to the secretion signal), were kept. Of the retained set, proteins containing a potential GPI-anchor signal as predicted by the GPIsom web service were discarded. Eventually, WoLF PSORT was used to estimate the located sites and only those proteins that were credibly positioned in the extracellular space (i.e., extracellular score >15) were included into in the final secretome. The secreted proteins were submitted to the Merops database (e-value = 1e-04) (Release 9.12; http://merops.sanger.ac.uk/) for predicting peptidases[42], and false positives were eliminated by parsing hits obtained following a BLASTp search (e-value = 1e-04) on the NCBI nr protein database. Small secreted proteins (SSPs) were defined here as the secreted proteins that are smaller than 200 aa, and an SSP was labeled as "cysteine-rich" when the percentage of cysteine residues in the protein was at least twice as high as the average percentage of cysteine residues in all predicted proteins of that species[43].

Secondary metabolism genes.

Nonribosomal peptide synthase (NRPS), polyketide synthase (PKS), hybrid NRPS-PKS,

dimethylallyl tryptophan synthase (DMATS), and terpene cyclase (TC) genes were identified in all 12 genomes by using the web-based software packages SMURF[44] and antiSMASH 2.0[45] in combination. Employing these two functionally similar methods simultaneously allowed us to detect small conflicts generated between their results, which were further checked and corrected. Another reason for using the packages in tandem is that although SMURF and antiSMASH alone are unable to predict TC and DMATS genes, they can complement each other for this purpose.

Supplementary References

1 Boetzer, M. & Pirovano, W. Toward almost closed genomes with GapFiller. *Genome Biol.* **13**, R56 (2012).

2 Tarailo- Graovac, M. & Chen, N. Using RepeatMasker to identify repetitive elements in genomic sequences. *Curr. Protoc. Bioinformatics* **25**, 4.10.11-14.10.14 (2009).

3 Jurka, J. *et al.* Repbase Update, a database of eukaryotic repetitive elements. *Cytogenet. Genome Res.* **110**, 462-467 (2005).

4 Holt, C. & Yandell, M. MAKER2: an annotation pipeline and genome-database management tool for second-generation genome projects. *BMC bioinformatics* **12**, 491 (2011).

5 Ter-Hovhannisyan, V., Lomsadze, A., Chernoff, Y. O. & Borodovsky, M. Gene prediction in novel fungal genomes using an ab initio algorithm with unsupervised training. *Genome Res.* **18**, 1979-1990 (2008).

6 Stanke, M. *et al.* AUGUSTUS: *ab initio* prediction of alternative transcripts. *Nucleic Acids Res.* **34**, W435-W439 (2006).

7 Korf, I. Gene finding in novel genomes. *BMC bioinformatics* **5**, 59 (2004).

8 Nordberg, H. *et al.* The genome portal of the Department of Energy Joint Genome Institute: 2014 updates. *Nucleic Acids Res.* **42**, D26-D31 (2014).

9 Parra, G., Bradnam, K., Ning, Z. M., Keane, T. & Korf, I. Assessing the gene space in draft genomes. *Nucleic Acids Res.* **37**, 289-297 (2009).

10 Gotz, S. *et al.* High-throughput functional annotation and data mining with the Blast2GO suite. *Nucleic Acids Res.* **36**, 3420-3435 (2008).

11 Altschul, S. F., Gish, W., Miller, W., Myers, E. W. & Lipman, D. J. Basic local alignment search tool. *J. Mol. Biol.* **215**, 403-410 (1990).

12 Ashburner, M. *et al.* Gene Ontology: tool for the unification of biology. *Nat. Genet.* **25**, 25-29 (2000).

13 Jones, P. *et al.* InterProScan 5: genome-scale protein function classification. *Bioinformatics* **30**, 1236-1240 (2014).

14 Bairoch, A. The ENZYME database in 2000. *Nucleic Acids Res.* **28**, 304-305 (2000).

15 Kanehisa, M., Goto, S., Kawashima, S., Okuno, Y. & Hattori, M. The KEGG resource for deciphering the genome. *Nucleic Acids Res.* **32**, D277-D280 (2004).

16 Marchler-Bauer, A. *et al.* CDD: NCBI's conserved domain database. *Nucleic Acids Res.* **43**, D222-226 (2015).

17 Finn, R. D. *et al.* Pfam: the protein families database. *Nucleic Acids Res.* **42**, D222-D230 (2014).

18 Tatusov, R. L., Galperin, M. Y., Natale, D. A. & Koonin, E. V. The COG database: a tool

for genome-scale analysis of protein functions and evolution. *Nucleic Acids Res.* **28**, 33-36 (2000).

19 Lagesen, K. *et al.* RNAmmer: consistent and rapid annotation of ribosomal RNA genes. *Nucleic Acids Res.* **35**, 3100-3108 (2007).

20 Richard, G. F., Kerrest, A. & Dujon, B. Comparative genomics and molecular dynamics of DNA repeats in eukaryotes. *Microbiol. Mol. Biol. R.* **72**, 686-727 (2008).

21 Li, L., Stoeckert, C. J. & Roos, D. S. OrthoMCL: Identification of ortholog groups for eukaryotic genomes. *Genome Res.* **13**, 2178-2189 (2003).

22 Enright, A. J., Van Dongen, S. & Ouzounis, C. A. An efficient algorithm for large-scale detection of protein families. *Nucleic Acids Res.* **30**, 1575-1584 (2002).

23 Katoh, K., Asimenos, G. & Toh, H. Multiple alignment of DNA sequences with MAFFT. *Methods Mol. Biol.* **537**, 39-64 (2009).

24 Castresana, J. Selection of conserved blocks from multiple alignments for their use in phylogenetic analysis. *Mol. Biol. Evol.* **17**, 540-552 (2000).

25 Darriba, D., Taboada, G. L., Doallo, R. & Posada, D. ProtTest 3: fast selection of best-fit models of protein evolution. *Bioinformatics* **27**, 1164-1165 (2011).

26 Stamatakis, A. RAxML-VI-HPC: Maximum likelihood-based phylogenetic analyses with thousands of taxa and mixed models. *Bioinformatics* **22**, 2688-2690 (2006).

27 Tamura, K., Stecher, G., Peterson, D., Filipski, A. & Kumar, S. MEGA6: Molecular Evolutionary Genetics Analysis Version 6.0. *Mol. Biol. Evol.* **30**, 2725-2729 (2013).

28 Padovan, A. C. B., Sanson, G. F. O., Brunstein, A. & Briones, M. R. S. Fungi evolution revisited: Application of the penalized likelihood method to a bayesian fungal phylogeny provides a new perspective on phylogenetic relationships and divergence dates of ascomycota groups. *J. Mol. Evol.* **60**, 726-735 (2005).

29 Felsenstein, J. Inferring phylogenies from protein sequences by parsimony, distance, and likelihood methods. *Method. Enzymol.* **266**, 418-427 (1996).

30 Yin, Y. B. *et al.* dbCAN: a web resource for automated carbohydrate-active enzyme annotation. *Nucleic Acids Res.* **40**, W445-W451 (2012).

31 Zhao, Z. T., Liu, H. Q., Wang, C. F. & Xu, J. R. Comparative analysis of fungal genomes reveals different plant cell wall degrading capacity in fungi. *Bmc Genomics* **14**, 274 (2013).

32 Rytioja, J. *et al.* Plant-Polysaccharide-Degrading Enzymes from Basidiomycetes. *Microbiol. Mol. Biol. R.* **78**, 614-649 (2014).

33 Kubicek, C. P., Starr, T. L. & Glass, N. L. Plant cell wall-degrading enzymes and their secretion in plant-pathogenic fungi. *Annu. Rev. Phytopathol.* **52**, 427-451 (2014).

34 Murphy, C., Powlowski, J., Wu, M., Butler, G. & Tsang, A. Curation of characterized glycoside hydrolases of fungal origin. *Database : the journal of biological databases and curation* **2011**, bar020 (2011).

35 Choi, J., Kim, K. T., Jeon, J. & Lee, Y. H. Fungal plant cell wall-degrading enzyme database: a platform for comparative and evolutionary genomics in fungi and Oomycetes. *Bmc Genomics* **14**, S7 (2013).

36 Petersen, T. N., Brunak, S., von Heijne, G. & Nielsen, H. SignalP 4.0: discriminating signal peptides from transmembrane regions. *Nat. Methods* **8**, 785-786 (2011).

37 Krogh, A., Larsson, B., von Heijne, G. & Sonnhammer, E. L. Predicting transmembrane

protein topology with a hidden Markov model: application to complete genomes. *J. Mol. Biol.* **305**, 567-580 (2001).

38 Emanuelsson, O., Nielsen, H., Brunak, S. & von Heijne, G. Predicting subcellular localization of proteins based on their N-terminal amino acid sequence. *J. Mol. Biol.* **300**, 1005-1016 (2000).

39 Fankhauser, N. & Maser, P. Identification of GPI anchor attachment signals by a Kohonen self-organizing map. *Bioinformatics* **21**, 1846-1852 (2005).

40 Horton, P. *et al.* WoLF PSORT: protein localization predictor. *Nucleic Acids Res.* **35**, W585-587 (2007).

41 Emanuelsson, O., Brunak, S., von Heijne, G. & Nielsen, H. Locating proteins in the cell using TargetP, SignalP and related tools. *Nat. Protoc.* **2**, 953-971 (2007).

42 Rawlings, N. D., Waller, M., Barrett, A. J. & Bateman, A. MEROPS: the database of proteolytic enzymes, their substrates and inhibitors. *Nucleic Acids Res.* **42**, D503 (2014).

43 Ohm, R. A. *et al.* Diverse lifestyles and strategies of plant pathogenesis encoded in the genomes of eighteen *Dothideomycetes* fungi. *PLoS Pathog.* **8**, e1003037 (2012).

44 Khaldi, N. *et al.* SMURF: Genomic mapping of fungal secondary metabolite clusters. *Fungal Genet. Biol.* **47**, 736-741 (2010).

45 Blin, K. *et al.* antiSMASH 2.0-a versatile platform for genome mining of secondary metabolite producers. *Nucleic Acids Res.* **41**, W204-W212 (2013).
